# Structural insight into the binding of human galectins to corneal keratan sulfate, its desulfated form and related saccharides

**DOI:** 10.1038/s41598-020-72645-9

**Published:** 2020-09-24

**Authors:** Michelle C. Miller, Chao Cai, Kanin Wichapong, Sayantan Bhaduri, Nicola L. B. Pohl, Robert J. Linhardt, Hans-Joachim Gabius, Kevin H. Mayo

**Affiliations:** 1 Department of Biochemistry, Molecular Biology & Biophysics, University of Minnesota, Minneapolis, MN 55455 USA; 2 Biocatalysis and Metabolic Engineering, Rensselaer Polytechnic Institute, Troy, NY 12180 USA; 3 Department of Biochemistry and the Cardiovascular Research Institute Maastricht (CARIM), Maastricht University, Maastricht, The Netherlands; 4 Department of Chemistry, Indiana University, Bloomington, IN 47405 USA; 5 Institute of Physiological Chemistry, Faculty of Veterinary Medicine, Ludwig-Maximillians-University Munich, 80539 Munich, Germany

**Keywords:** Biochemistry, Biophysics

## Abstract

Glycosaminoglycan chains of keratan sulfate proteoglycans appear to be physiologically significant by pairing with tissue lectins. Here, we used NMR spectroscopy and molecular dynamics (MD) simulations to characterize interactions of corneal keratan sulfate (KS), its desulfated form, as well as di-, tetra- (*N*-acetyllactosamine and lacto-*N*-tetraose) and octasaccharides with adhesion/growth-regulatory galectins, in particular galectin-3 (Gal-3). The KS contact region involves the lectin canonical binding site, with estimated K_D_ values in the low µM range and stoichiometry of ~ 8 to ~ 20 galectin molecules binding per polysaccharide chain. Compared to Gal-3, the affinity to Gal-7 is relatively low, signaling preferences among galectins. The importance of the sulfate groups was delineated by using desulfated analogs that exhibit relatively reduced affinity. Binding studies with two related di- and tetrasaccharides revealed a similar decrease that underscores affinity enhancement by repetitive arrangement of disaccharide units. MD-based binding energies of KS oligosaccharide-loaded galectins support experimental data on Gal-3 and -7, and extend the scope of KS binding to Gal-1 and -9N. Overall, our results provide strong incentive to further probe the relevance of molecular recognition of KS by galectins in terms of physiological processes in situ, e.g. maintaining integrity of mucosal barriers, intermolecular (lattice-like) gluing within the extracellular meshwork or synaptogenesis.

## Introduction

As a key aspect of their (patho)physiological functionality, the sugar code concept ascribes the nature of molecular messages to glycans that are ‘read’ and ‘translated’ into cellular effects by tissue lectins^1–7^. Therefore, it is reasonable to assume that pairing up to a local network of binding partners at sites of preferential presence of a distinct glycoconjugate will likely occur. When having disclosed such well-structured patterns of expression for glycosaminoglycans by bio- and histochemical mapping, the characterization of modes of molecular recognition with tissue lectins is warranted as the next step toward clarifying the issue on functional significance, both for the lectin and for the glycan. Here, we exemplify this hypothesis-driven approach by analyzing the interaction between bovine corneal keratan sulfate (KS) with human adhesion/growth-regulatory lectins present in the cornea, i.e. galectin-3 (Gal-3) and Gal-7, by using NMR spectroscopy and also molecular dynamics (MD) simulations with two other galectin family members present in the cornea (Gal-1 and Gal-9) to depict bound-state conformation(s) and to calculate and compare binding energies.

KS was first discovered in extracts from cornea, where it is abundantly present^8^. It was biochemically defined as “sulfated mucopolysaccharide, composed of equimolar quantities of glucosamine, acetyl, galactose, and sulfate, for which we propose the name keratosulfate”^9^. In structural terms, KS belongs to the family of glycosaminoglycans: its disaccharide building block *N*-acetyllactosamine (LacNAc, type II) can be sulfated at the galactose C6′ position and the *N*-acetylglucosamine (GlcNAc) C6 position^10–16^. The “sugar code” for KS is relatively simple compared to heparin, heparan sulfate, chondroitin sulfate and dermatan sulfate, due to its more homogeneous sulfation pattern that is basically composed of the four major disaccharides (Gal-GlcNAc, Gal6S-GlcNAc, Gal-GlcNAc6S, and Gal6S-GlcNAc6S). This leads to fewer micro-heterogeneities in the KS chain compared to those from other sulfated GAGs and therefore, much lower variations for protein interactions (i.e. a small sugar code). Thus, positional diversity of sulfation is less than that seen in e.g. heparan sulfate, directing interest to the backbone as the contact site. With respect to possible pairing partners, it is inspiring that the sulfate-free disaccharide unit is the canonical ligand for the family of ga(lactose-binding)lectins that are broad-impact effectors in cell physiology by bridging (in *trans* and in *cis*) glycoconjugate-based counter-receptors^17–20^. Providing substantial incentive for our study is the report that 6-*O*-sulfated LacNAc and a trisulfated LacNAc dimer (structural units in corneal KS) are indeed potent ligands for galectins, as detected for Gal-1, -3 and -9^21–23^.

Giving our study a clear direction is a comparative analysis within the galectin family that has identified Gal-3 as a major binding partner for KS^22^, which is fitting for an in situ interaction where expression of this lectin is strong and phylogenetically conserved in cornea^24–28^. In addition, Gal-3 is known: (i) to bind to LacNAc oligomers with relatively high affinity that increases with chain length^29–34^ and, as a hint to functional interplay, (ii) to contribute to the integrity of the ocular surface epithelial barrier^35^. Cross-linking spatially accessible polyLacNAc chains by a bi- or oligo-valent galectin that then acts like a molecular glue, can thus provide meaning to the development of the respective enzymatic machinery for this type of glycosaminoglycan^16,36,37^ that may help explain local co-expression of KS-presenting proteoglycans and galectins. Since other members of the galectin family with cross-linking capacity are also present in the corneal epithelium (in particular Gal-7 and -9, with the latter being upregulated in cornea-infected by *P. aeruginosa*^26,27,38^), we broadened the scope of our study beyond Gal-3 to include these galectins as well.

To advance from simply detecting interactions between galectins and KS (and its desulfated form KSDS) to elucidating molecular details of their binding, we performed [^15^N–^1^H] HSQC NMR studies with ^15^N-labeled Gal-3 [full-length (FL) and its carbohydrate recognition domain (CRD)] and Gal-7 with KS and KSDS as ligands. It is noteworthy that KSDS resembles embryonic KS prior to the onset of developmental sulfation^39,40^ that is a potential docking site for developmentally expressed galectins as noted in work on the anterior segment of the adult chicken eye^28^. Mapping the galectin-binding epitope was also performed using compounds akin to the building blocks of KS, i.e. the disaccharide LacNAc and the tetrasaccharide lacto-*N*-tetraose (LNT). To extend this empirical approach, we also ran MD simulations with KS-based oligosaccharides (di-, tetra- and octasaccharides) in order to visualize the contact profile and to facilitate calculation of binding energies. Due to the well-defined capacity to accommodate polyLacNAc chains by the N-terminal CRD of human Gal-9 (Gal-9N^41^) and the detection of different contact sites for Gal-1 (terminal) vs Gal-3 (internal) on these oligosaccharides^32,33,42^, we also investigated Gal-1 and -9N using the same approach.

## Results and discussion

### KS binding to Gal-3 assessed by NMR

**Figure 1:**
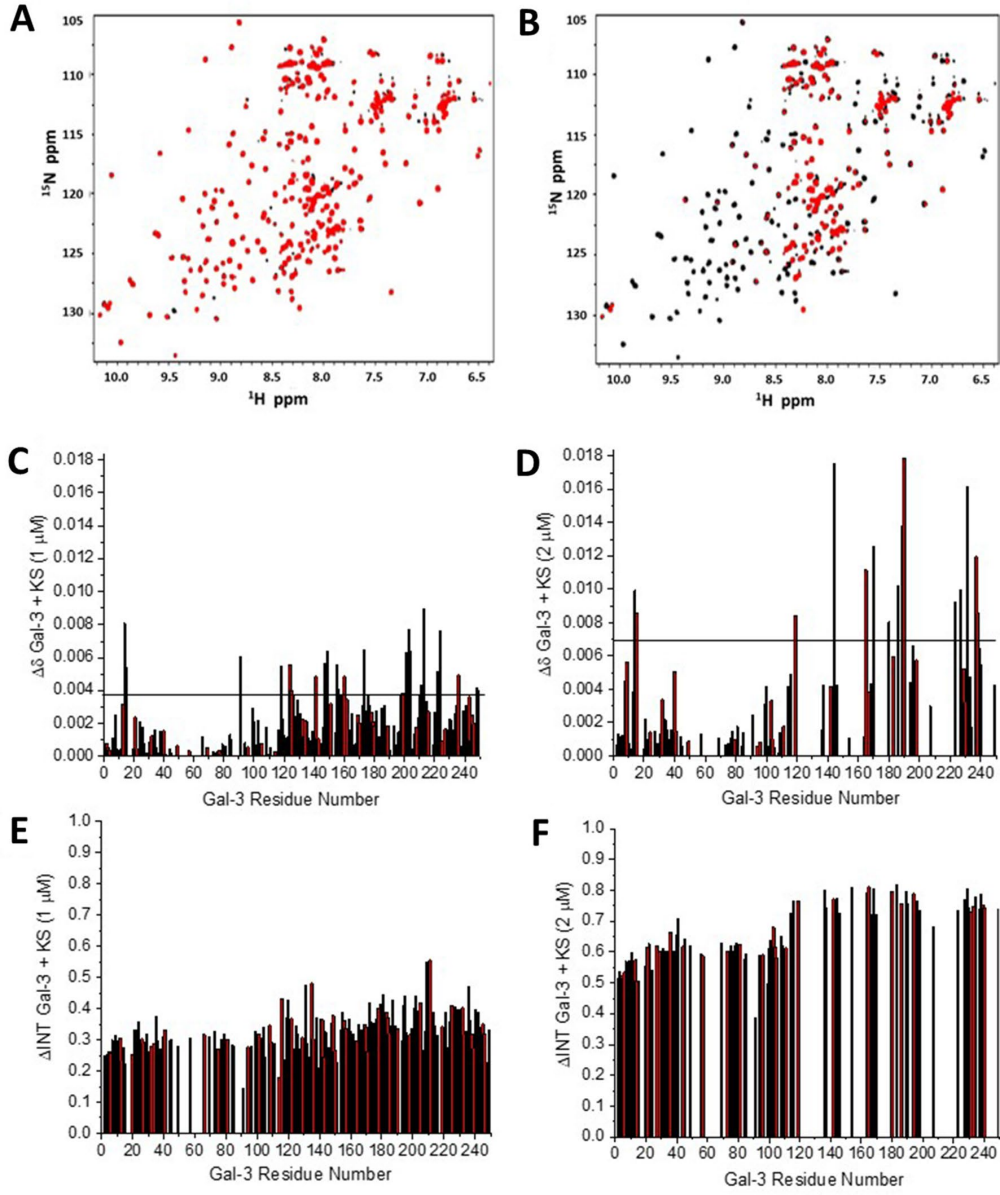
HSQC data of full-length Gal-3 in complex with KS. ^1^H–^15^N HSQC spectra for ^15^N-enriched Gal-3 FL alone (20 μM, peaks in black) and in the presence of keratan sulfate (KS, red peaks) at 1 μM (**A**) and 2 μM (**B**) are shown. Solution conditions are 20 mM KPhos, pH 6.9, 30 °C. HSQC chemical shift maps (∆δ vs. amino acid sequence) are shown for the binding of Gal-3 FL to KS at 1 μM (**C**) and 2 μM; the horizontal black line indicates 1SD above the average value (**D**). Resonance broadening maps (∆Intensity or ∆INT vs. amino acid sequence) are shown, respectively, in (**E**) and (**F**). A value of 1 indicates that the resonance obtained from that particular residue is no longer detectable, and a value of zero indicates no change in resonance intensity.

HSQC data analysis is often used to determine site-specific ligand-receptor interactions and titration-based binding affinities. Figure 1A,B shows HSQC spectra of Gal-3 FL (20 μM) in the absence (black peaks) and presence (red peaks) of KS. Upon addition of 1 μM KS (Fig. 1A), various resonances are highly reduced in intensity (i.e. broadened) and minimally chemically shifted. At 2 μM KS (Fig. 1B), the extent of these spectral changes has increased.

In NMR ligand-binding studies, interactions between ligand and receptor occur via an NMR frequency-dependent exchange between bound and free states. On the chemical shift time scale, NMR exchange regimes range from fast (K_D_ >  ~ 100 μM), to slow (K_D_ <  ~ 1 μM), and in between, i.e. intermediate (K_D_ ~ 2 μM to ~ 100 μM)^43^. Fast exchange is reflected in smoothly shifting resonances as weighted populations between bound and unbound states vary during the titration. Slow exchange is indicated when two (or more) resonances are simultaneously observed with relative intensities depending on the weighted populations of bound and unbound. With intermediate exchange, resonances are minimally chemically shifted and significantly decreased in intensity (i.e. broadened) as populations vary from free to bound. Intermediate exchange includes changes in internal motions, bound-free dynamics, oligomer state exchange. Our HSQC data indicate that interactions between Gal-3 and KS occur in the intermediate exchange regime with affinities/avidities fall in the μM range^43^.

Chemical shift maps (∆δ vs. Gal-3 sequence) for KS concentrations of 1 μM and 2 μM are shown in Fig. 1C,D, respectively, along with resonance broadening maps (∆Intensity vs. Gal-3 sequence) in Fig. 1E,F. At 1 μM KS, most of the shifted resonances belong to residues within the canonical sugar-binding S-face of the lectin (Fig. 1C). In addition, residues within the opposing F-face of the CRD are also affected, as well as regions within the N-terminal tail (NT). At 2 μM KS, many of the protein’s resonances are so much broadened as to be no longer observed (Fig. 1F), thus severely limiting the information that chemical shift maps offer (Fig. 1D). ^15^N–^1^H HSQC spectra also contain information on ^15^N-labeled side chains of asparagine and glutamine, and sometimes arginine, residues that are involved in binding interactions with sugars at the CRD canonical site. Supplemental Figure S1 shows ∆δ and ∆Intensity values for those side chain resonances that could be observed, i.e. asparagine and glutamine. Unfortunately, the ^15^N ppm range in our HSQC spectra was not broad enough to directly capture arginine Nε–Hε side chain resonances, and spectral fold overs from these arginine resonances that can sometimes be observed, only showed a few of them with relatively weak intensities in free and KS-bound states that could not be interpreted accurately. Nonetheless, ∆δ values for the asparagine and glutamine side chain resonances within the canonical sugar binding S-face are generally larger than those from the backbone, consistent with interactions upon KS binding occurring with these side chains.

**Figure 2:**
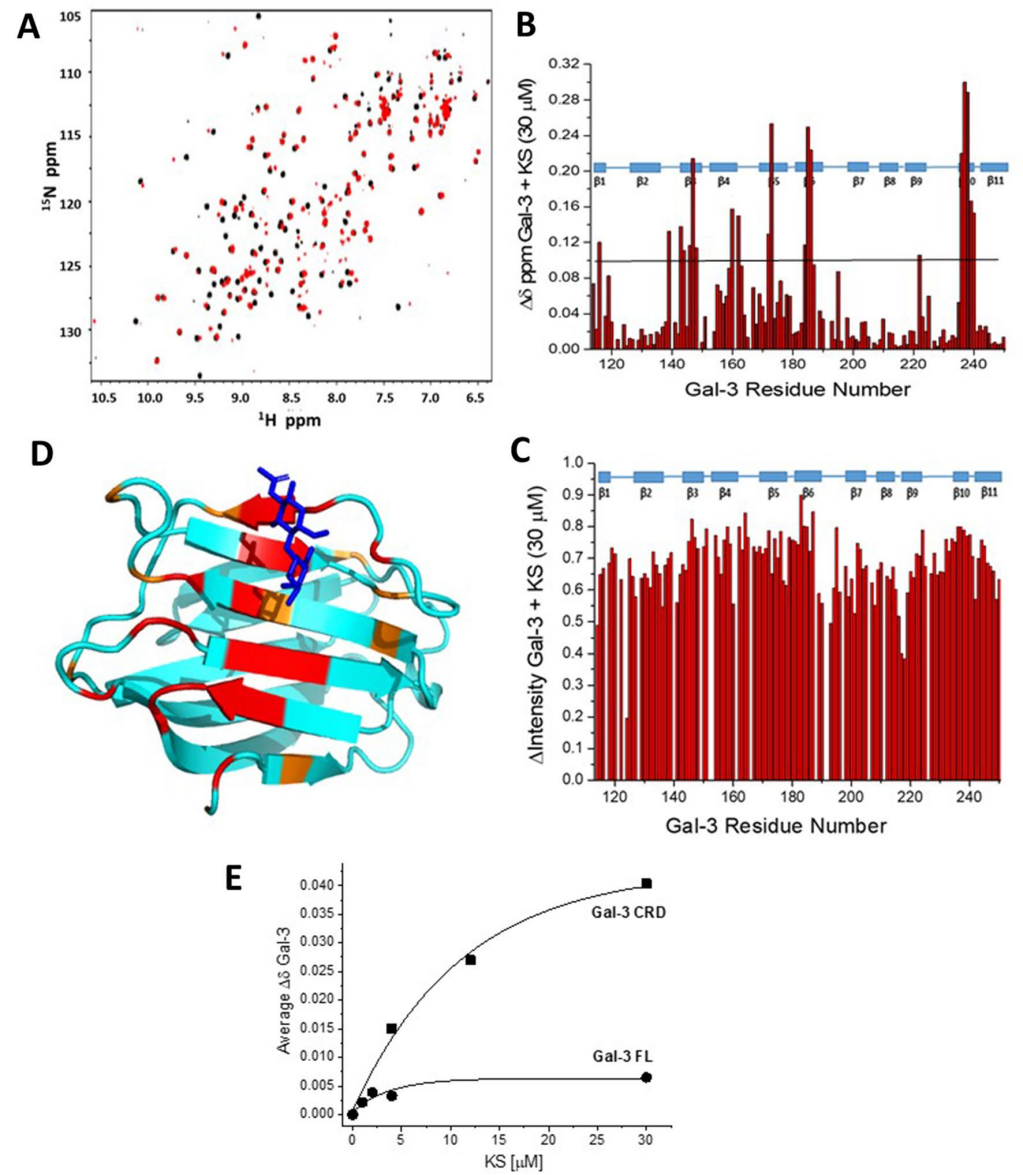
HSQC data of Gal-3 CRD in complex with KS. ^1^H–^15^N HSQC spectrum of ^15^N-enriched Gal-3 CRD alone (20 μM, peaks in black) and in the presence of 30 μM KS (**A**). Solution conditions are 20 mM KPhos, pH 6.9, 30 °C. The chemical shift map (∆δ vs. amino acid sequence with the horizontal black line indicating 1SD above the average value) and resonance broadening map (∆Intensity or ∆INT vs. amino acid sequence) derived from the HSQC spectrum presented in (**A**) are shown in (**B**) and (**C**) (positioned below each other), respectively. In the broadening map, a value of 1 indicates that the resonance obtained from that particular residue is no longer detectable, and a value of zero indicates no change in resonance intensity. (**D**) Residues most perturbed by Gal-3 CRD binding to KS are highlighted in red (2 SD above the average ∆δ value) and orange (1 SD above the average ∆δ value) on the structure of the Gal-3 CRD (PDB 1A3K), as discussed in the text. All others with SD < 1 are colored in cyan. A bound lactose molecule is shown in blue. (**E**) The average ∆δ values from Gal-3 FL and Gal-3 CRD HSQC spectra are plotted vs. the KS concentration (μM). Solid lines show exponential fits to these data.

The problem with extreme line broadening from KS-loaded full-length Gal-3 prompted us to investigate KS binding to Gal-3 CRD that is devoid of the NT. Indeed, a clearer picture of Gal-3:KS interactions resulted by our monitoring the binding of KS to the Gal-3 CRD (Fig. 2). Figure 2A shows an HSQC spectrum of the Gal-3 CRD (20 μM) in the absence (black peaks) or presence (red peaks) of KS at 30 μM where spectral effects are near maximal. Compared to Gal-3 FL, KS-induced resonance shifts are larger, occur more smoothly, and peak at a higher KS concentration. The chemical shift (and broadening) maps (Fig. 2B,C) underscores that the contact occurs within the (canonical) S-face of the CRD, as illustrated in the color-highlighted structure of Gal-3 CRD (Fig. 2D). In addition to perturbing residues primarily within strands β3, β4, β5, and β6, like the disaccharide lactose does (see survey of NMR-derived information on Gal-3^44^), binding of KS, and less so of KSDS, also has a significant effect on residues within strands β1 and β10 at the base of the S-face β-sheet. This observation is consistent with a larger binding footprint of the glycosaminoglycan chain on the Gal-3 CRD than that of a disaccharide. It is noteworthy that several charged residues at the right side of the S-face (see Fig. 2E) are minimally shifted, if at all, suggesting that KS interactions with Gal-3 prefer residues at the left side of S-face. Obviously, this contact pattern lets other functionally active regions remain accessible, e.g. for binding chemokines like CXCL12^45^ or other galectin CRDs for heterodimerization^46^.

These data also indicate that interactions between the Gal-3 CRD and KS occur more towards the fast exchange regime on the chemical shift time scale, indicating that binding is weaker for the CRD than with Gal-3 FL. In order to comparatively illustrate data sets, Fig. 2E plots average chemical shift changes in Gal-3 FL and Gal-3 CRD as a function of the KS concentration. The 50% change in chemical shift occurs at about 2 μM for Gal-3 FL and at about 7 μM for Gal-3 CRD. This affinity/avidity might be altered by changing the status of sulfation, a structural hallmark of glycosaminoglycans that has implications for receptor association^47–50^. In principle, two opposite effects may result: enhanced affinity by unmasking the galactose moiety via removal of the 6′-sulfate group and reduced affinity by turning 6-sulfated GlcNAc into unsubstituted GlcNAc. To see what actually happens with galectin binding, we tested KS after desulfation (termed KSDS) under identical conditions.

Glycan binding-induced Gal-3 oligomerization has been reported with asialofetuin^55^ and lacto-*N*-neo-tetraose, LNnT^56^. Therefore, to assess the possibility of KS binding-induced galectin-galectin interactions or oligomerization that could indirectly reduce HSQC ^15^N-Gal-3 resonance intensities during KS titrations, we used pulsed field gradient (PFG) NMR to determine diffusion coeifficients, *D*, as a function of the Gal-3/KS molar ratio. For this, we focused on the most intense ^1^H resonances from KS (~ 3.64 ppm and 3.7 ppm, Supplemental Fig. S2) and monitored the effect from PFG-induced signal intensities as the Gal-3 concentration was increased. Supplemental Fig. S3 plots *D* values vs the Gal-3/KS molar ratio for full-length Gal-3 (filled squares) and for Gal-3 CRD (open circles). The *D* value for KS (~ 14,300 kDa) in the absence of Gal-3 is 0.92 × 10^–6^ cm^2^/s^-1^. If KS had a globular fold, *D* would theoretically (Stokes–Einstein model) be ~ 1.5 × 10^–6^ cm^2^/s^−1^. Thus, this lower *D* value indicates that KS, not unexpectedly, has a non-globular, extended and/or dynamic structure and/or self-associates to some extent.

Addition of either species of Gal-3 lowers the KS *D* value, indicating binding of the lectin to KS, and thus increasing the overall size of the complex. The effect is somewhat greater for full length Gal-3 due to its greater mass vis-à-vis Gal-3 CRD. Upon addition of one mole equivalent of Gal-3, the KS *D* value is decreased by about 40%, consistent with approximate doubling of the mass. In both instances, this trend continues until a Gal-3/KS molar ratio of ~ 6 to 8 is reached, suggesting a binding stoichiometry of up to ~ 8 Gal-3 molecules per KS molecule. If Gal-3 were oligomerizing upon binding to KS, these *D* values would be considerably smaller (i.e. increased mass), and they are not, indicating that KS-induced Gal-3 oligomerization, if it does occur, does not do so to any significant extent. In addition, at Gal-3/KS ratios of 15 and 20, *D* values increase somewhat. Even though this increase in *D* values could be explained by Gal-3 binding to KS and reduction of any KS–KS self-association as reported for Gal-1 binding to a rhamnogalaturonan polysaccharide^51^, this effect should occur near the beginning of the titration and not at the end. Therefore, it is most likely that ligand-free Gal-3 simply contributes to PFG-induced changes in resonance intensities (and thus increases *D* values) at higher concentrations of Gal-3. Although we cannot absolutely exclude KS binding-induced Gal-3 oligomerization, the trends in these *D* values indicate that it does not occur to any significant extent with KS.

### Sulfate groups modulate KS binding

**Figure 3:**
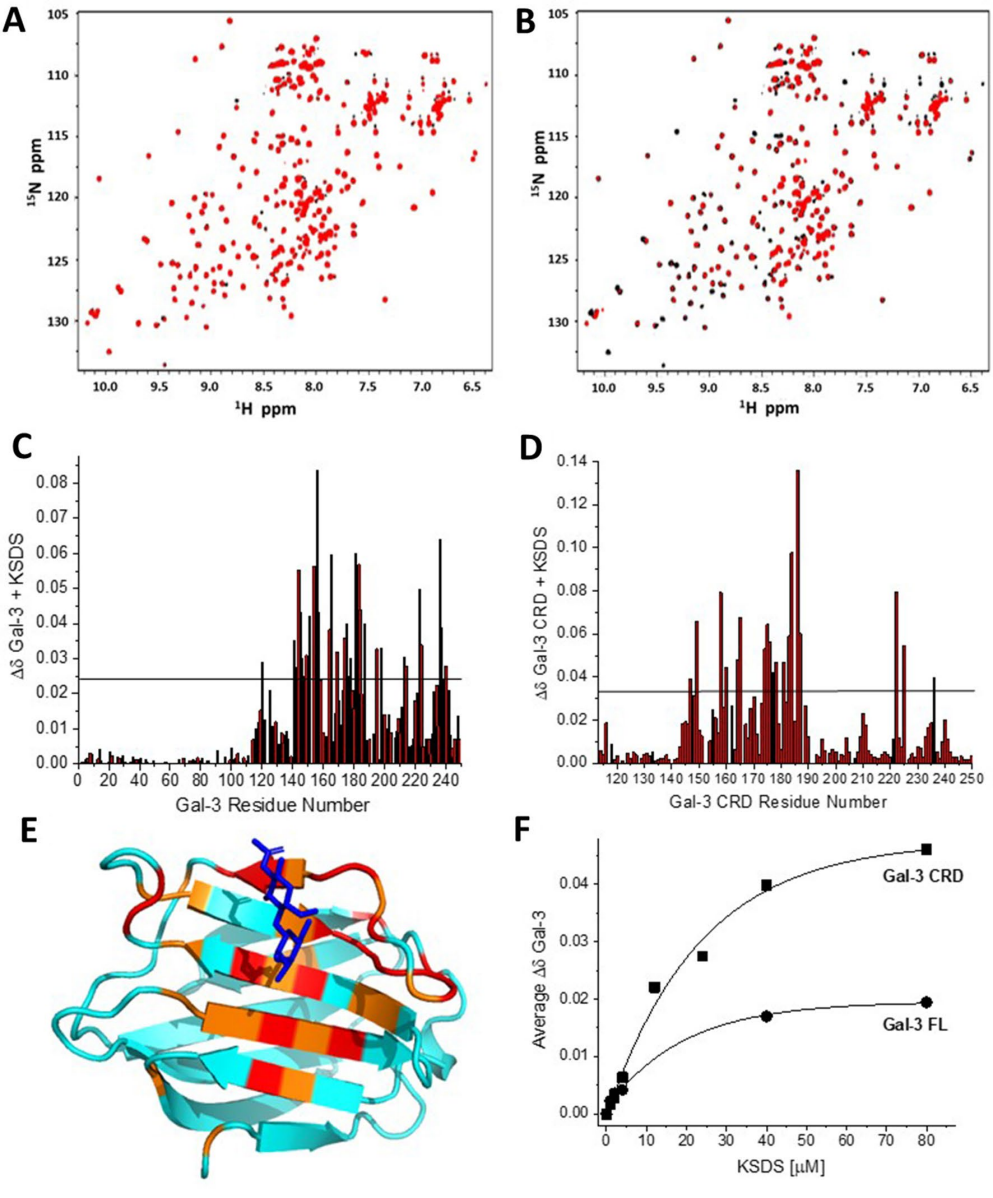
HSQC data of Gal-3 in complex with KSDS. ^1^H–^15^N HSQC spectra of ^15^N-enriched Gal-3 FL alone (20 μM, peaks in black) and in the presence of desulfated KSDS at 1 μM (**A**) and 2 μM (**B**) are shown. Solution conditions are 20 mM KPhos, pH 6.9, 30 °C. The chemical shift map (∆δ vs. amino acid sequence) for the binding of KSDS to Gal-3 FL is shown in (**C**), and that for the binding of KSDS to Gal-3 CRD is shown in (**D**). Horizontal black lines indicate 1SD above the average value. (**E**) Residues most perturbed by binding of Gal-3 CRD to KSDS are highlighted in red (2 SD above the average ∆δ value) and orange (1 SD above the average ∆δ value) on the structure of the Gal-3 CRD (PDB 1A3K), as discussed in the text. A lactose molecule associated to the canonical contact region of the CRD is shown in blue. (**E**) The average ∆δ values from Gal-3 FL and Gal-3 CRD HSQC spectra are plotted vs. the KSDS concentration (μM). Solid lines show exponential fits to these data.

Figure 3 shows HSQC spectra of Gal-3 FL in the absence and presence of KSDS at 1 μM (Fig. 3A) and 2 μM (Fig. 3B) for direct comparison to HSQC spectra of the lectin with KS at the same concentrations (Fig. 1A,B, respectively). Note here that spectral effects are less prominent with KSDS as the ligand than with KS. The same is observed with Gal-3 CRD and KSDS as ligand (data not shown). Chemical shift maps show that KSDS binds both Gal-3 FL (Fig. 3C, 40 μM KSDS) and Gal-3 CRD (Fig. 3D, 80 μM KSDS) essentially in the same way as does KS. This is illustrated for KSDS in Fig. 3E, which shows the structure of Gal-3 CRD with colors highlighting the most affected chemically shifted residues. Moreover, KSDS binding affinities/avidities are less than those with KS, i.e. 50% lectin bound to KSDS at 11 μM for Gal-3 FL and 17 μM and Gal-3 CRD. With KS binding, 50% change in chemical shift was observed at about 2 μM for Gal-3 FL and at about 7 μM for Gal-3 CRD. In Fig. 3F, chemical shift changes for Gal-3 CRD are overall larger than for Gal-3 FL, primarily because average ∆δ values are shown, and changes within the NT of Gal-3 FL are very small.

Aside from the binding of KSDS being comparatively weaker, the smaller NT and F-face changes observed upon binding of KSDS indicate that the presence of sulfate groups in KS induces more significant effects on residues within the F-face and NT. Moreover, because KS and KSDS target the S-face of Gal-3 CRD, spectral perturbations at F-face residues in Gal-3 FL likely result from changes in interactions between the CRD F-face and the NT, as opposed to direct binding of KS or KSDS to the F-face. Such a scenario had been reported previously for β-glucans^51^ and α-mannans^52^. Interestingly, binding of a bulky ligand to the S-face may affect the dynamics of NT backfolding, known to occur in solution^44^. Having herewith mapped the profile of events by KS/KSDS binding for Gal-3 using HSQC analysis, we next investigated their binding to the proto-type (homodimeric) Gal-7.

### Gal-7 also binds to KS

**Figure 4:**
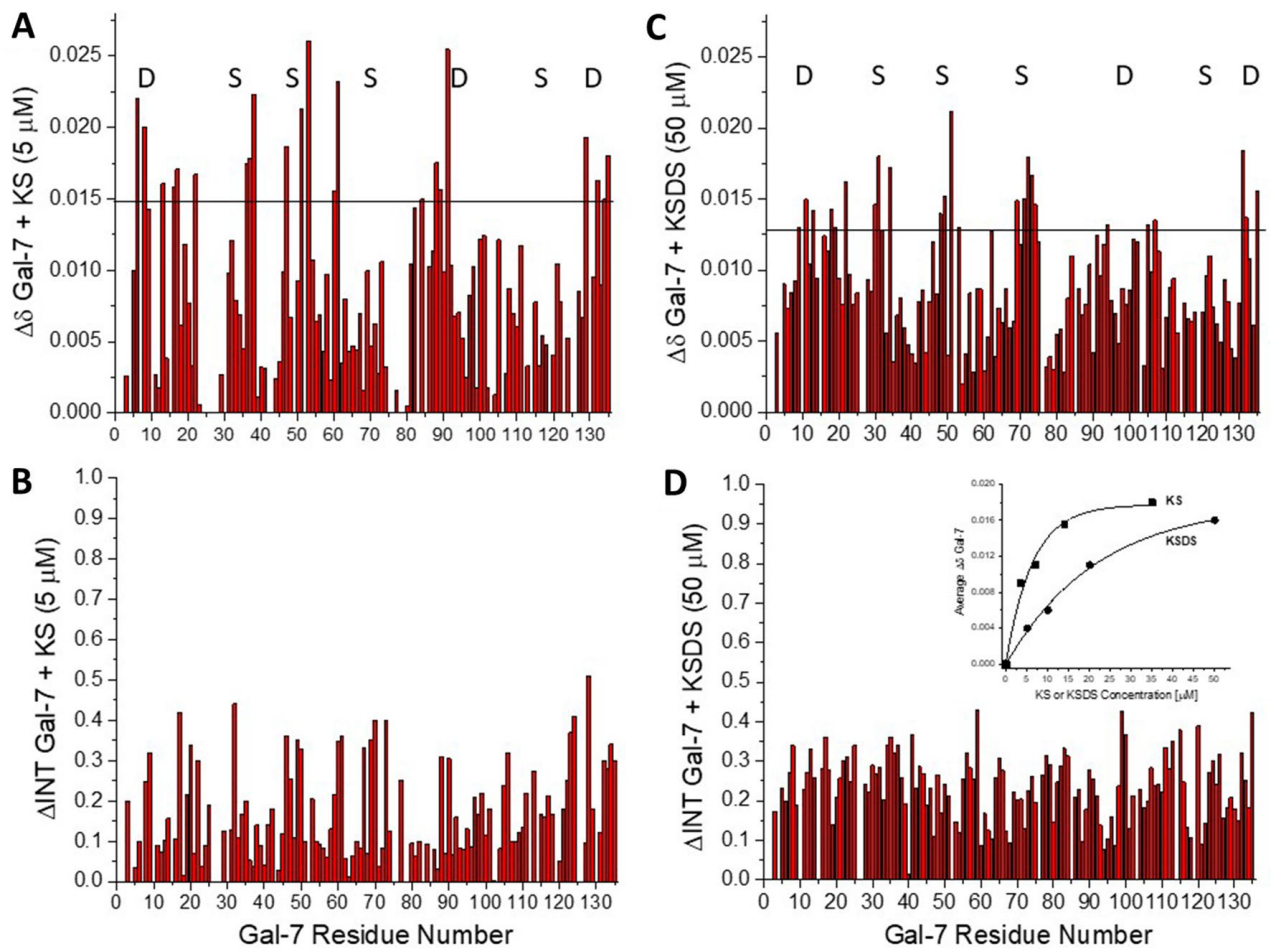
Chemical shift and broadening maps for KS and KSDS binding to Gal-7. Chemical shift (∆δ vs. amino acid sequence with the horizontal black line indicating 1SD above the average value) and resonance broadening (∆Intensity or ∆INT vs. amino acid sequence) maps derived from HSQC spectra of ^15^N-labeled Gal-7 (20 μM) (not shown) are presented in (**A**) and (**B**) in the presence of KS (5 μM) to Gal-7, and in (**C**) and (**D**) in the presence of KSDS (50 μM) to Gal-7. In the broadening maps, a value of 1 indicates that the resonance associated with that particular residue is no longer detectable, and a value of zero indicates no change in resonance intensity. (Insert to Fig. **D**) The average ∆δ values from HSQC of ^15^N-labeled Gal-7 are plotted vs. the KS or the KSDS concentration (μM) as indicated. Solid lines show exponential fits to these data.

HSQC spectra of ^15^N-labeled Gal-7 in the absence (black peaks) and/or presence of 5 μM of KS and of KSDS (red peaks) are shown in Supplemental Fig. S4A,B, respectively, and at 35 μM (KS) and 50 μM (KSDS) in Supplemental Fig. S4C,D, respectively. At either concentration, spectral effects are larger with KS than with KSDS. Figure 4 quantifies these data to provide an overview on what occurs. At 5 μM KS, the chemical shift and broadening changes are relatively small (Fig. 4A,B), yet greater than those with KSDS. In fact, one has to go ten-fold higher with KSDS (50 μM) to observe effects of similar magnitude (Fig. 4C,D). Gal-7 binding avidity can be estimated from plots of average ∆δ vs. glycosaminoglycan concentration (insert to Fig. 4D). With KS, 50% Gal-7 is loaded with ligand at ~ 4.5 μM, whereas 50% Gal-7 is saturated at ~ 15 μM by KSDS.

In terms of assessing binding characteristics of Gal-7 to KS or KSDS, we found that analysis of HSQC data is more complicated with Gal-7 than with Gal-3. Notice in Fig. 4A,C that there are no clear Gal-7 sequences where chemical shift changes are greatest. Spectral perturbations occur throughout both S- and F-faces (Fig. 4A,C). If we focus on the sugar-binding S-face, we can see that a set of Gal-7 residues appears to interact with KS and KSDS (Fig. 4A,C labeled with the letter “S”) in a similar fashion as with Gal-3. Other equally significant shift perturbations with Gal-7, however, are observed at residues within its CRD F-face. The most likely explanation for this is that ligand binding at the canonical S-face of Gal-7 allosterically affects residues at the F-face which forms the Gal-7 dimer interface (PDB 4GAL; Fig. 4A,C labeled with the letter “D”), thus perturbing Gal-7 dimer formation. In fact, lactose binding (at the canonical site) has previously been reported to trigger respective structural changes^53^. This finding prompted us to analyze interactions between a di- and a tetrasaccharide (that represent structural units of KSDS) and Gal-3 CRD, as well as Gal-7.

### Gal-3 and Gal-7 binding to LacNAc and LNT

**Figure 5:**
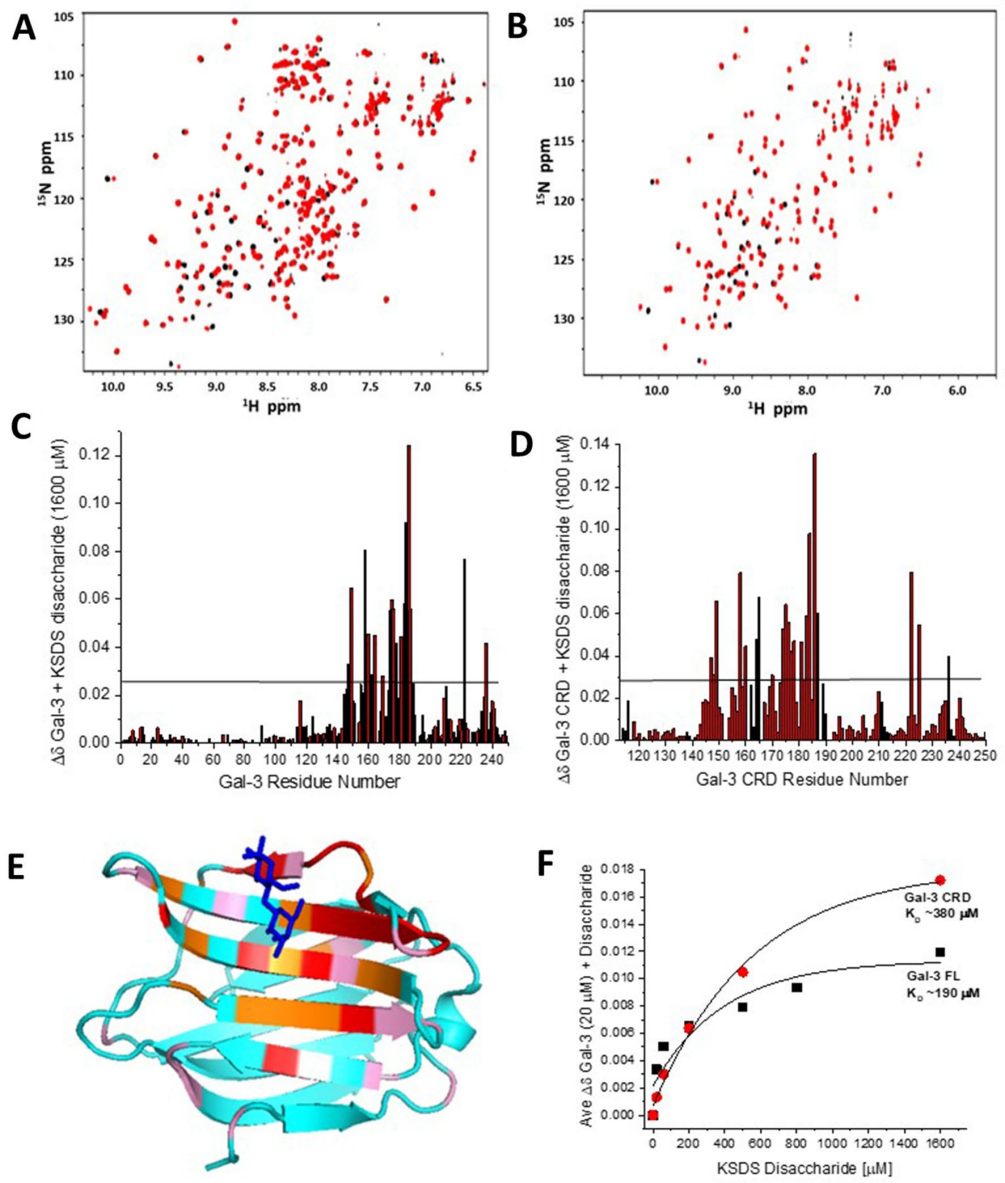
HSQC spectra of Gal-3 in complex with the disaccharide unit (LacNAc) of KSDS. ^1^H–^15^N HSQC spectra are shown for 20 μM ^15^N-enriched Gal-3 FL (**A**) or Gal-3 CRD (**B**) alone (peaks in black) and in the presence of 1.6 mM disaccharide unit of KSDS. Solution conditions are 20 mM KPhos, pH 6.9, 30 °C. Chemical shift maps (∆δ vs. amino acid sequence) from data in (**A**) and (**B**) are shown in (**C**) and (**D**), respectively. Horizontal black lines indicate 1SD above the average value. (**E**) Residues of Gal-3 CRD most perturbed by binding to LacNAc are highlighted in red (2 SD above the average ∆δ value) and orange (1 SD above the average ∆δ value) on the structure of the Gal-3 CRD (PDB 1A3K), as discussed in the text. A bound lactose molecule is shown in blue. (**F**) The average ∆δ values from Gal-3 FL and Gal-3 CRD HSQC spectra are plotted vs. the KSDS disaccharide concentration (μM). Solid lines show exponential fits to these data.

HSQC spectra of Gal-3 FL and Gal-3 CRD in the absence (black peaks) or presence (red peaks) of 1.6 mM LacNAc are shown in Fig. 5A,B, respectively. In either case, many peaks are shifted significantly. Chemical shift maps of each (Fig. 5C,D, respectively) show that the largest changes are within the canonical sugar-binding S-face of the CRD, as illustrated with color highlights on the structure of the Gal-3 CRD (PDB 1A3K; Fig. 5E). It is noteworthy that unlike Gal-3 binding to KSDS, the extent of chemical shift changes of residues within the NT and F-face is now relatively small, if at all present, indicating minimal perturbations by direct contact or by allosteric effects within either non-canonical segment induced by ligand binding. Moreover, because the association occurs in the fast exchange regime on the chemical shift time scale, K_D_ values can be derived fairly accurately from the dependence of chemical shifts on ligand concentration (Fig. 5F). This dependence also offers a means to estimate binding stoichiometry.

Using these data, LacNAc is calculated to bind to Gal-3 FL and CRD with K_D_ values of 190 μM and 380 μM, respectively. If we assume that a KSDS chain forms a complex with more than a single Gal-3 protein, we can use these K_D_ values and the 50% bound mark with the glycosaminoglycan to estimate binding stoichiometry to KSDS. In this regard, about 17 binding sites (190 μM/11 μM) in the KSDS for Gal-3 FL and about 22 binding sites (380 μM/17 μM) for Gal-3 CRD can be estimated. If binding stoichiometries for Gal-3 in KS are similar, then we would have a site-average microscopic K_D_ of 34 μM (= 2 μM × 17 sites) for Gal-3 FL and 154 μM (= 7 μM × 22 sites) for Gal-3 CRD. Of course, there would be fewer binding sites on each chain if per-site binding affinities were higher, i.e. stronger binding, and even though these stoichiometries are only estimates, they are consistent with the observed spectral perturbation differences for the two Gal-3 species when interacting with KS or KSDS. Nevertheless, ~ 20 Gal-3 binding sites on each molecule of KS or KSDS seems unrealistically high, given that the length of either polysaccharide is ~ 50 residues and filling the CRD sugar binding site would take up ~ 5 residues/Gal-3, a more realistic binding stoichiometry is < 10:1, as suggested by our PFG NMR diffusion results. Furthermore, because the glycos-aminoglycan is polyvalent for a galectin, as are glycoproteins with *N*- or mucin-type core 2/4 *O*-glycans^54^, it is possible that the degree of saturation affects the affinity at each step. In order to delineate a gradient of the affinity constant with progressively loading of binding sites (as reported for *N*-glycan binding of a nona-valent glycoprotein (asialofetuin) by human galectins^55^), titration calorimetry covering the full range up to saturation would be informative. Notably, in that case, the first binding event(s) will have a conspicuously high affinity, which may reflect physiological significance.

**Figure 6:**
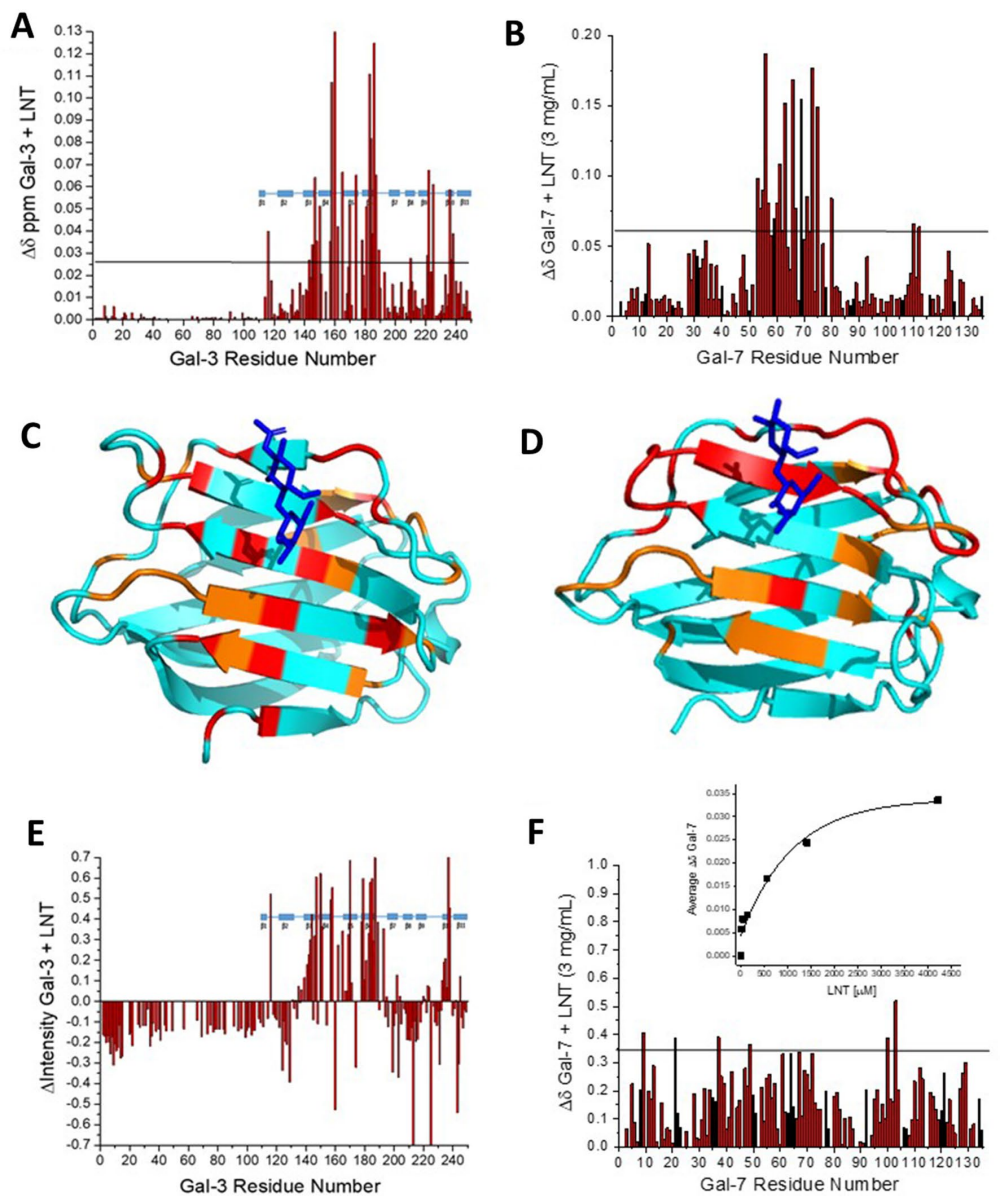
HSQC data for Gal-3 and Gal-7 binding to the tetrasaccharide LNT. (**A,B**) Chemical shift maps (∆δ vs. amino acid sequence) derived from HSQC spectra of ^15^N-labeled Gal-3 FL (20 μM) and ^15^N-labeled Gal-7 (not shown) in the presence of 4.2 mM LNT are provided in (**A**) and (**B**), respectively. Horizontal black lines indicate 1SD above the average value. (**C,D**) Residues whose chemical shifts are most perturbed by LNT binding to Gal-3 FL (**C**) and Gal-7 (**D**) are highlighted in red (2 SD above the average ∆δ value) and orange (1 SD above the average ∆δ value) on the structures of the Gal-3 CRD (PDB 1A3K) or Gal-7 CRD (PDB 4GAL), as discussed in the text. Bound molecules of lactose or LNT are shown in blue in (**C**) and (**D**), respectively. (**E,F**) Resonance broadening maps (∆Intensity or ∆INT vs. amino acid sequence) associated with these data are shown in (**E**) and (**F**), respectively. In the broadening map, a value of 1 indicates that the resonance obtained from that particular residue is no longer detectable, and a value of zero indicates no change in resonance intensity. (Insert to **F**) Average ∆δ values for the binding of LNT to Gal-7 are plotted vs. the LNT concentration (μM). Solid lines show exponential fits to these data. Solution conditions are 20 mM KPhos, pH 6.9, 30 °C.

Figure 6A,B shows that LNT binds to Gal-3 FL and to Gal-7 at their canonical sugar-binding S-faces, as illustrated by the largest ∆δ values color highlighted on the crystal structures of Gal-3 CRD (PDB 1A3K) and Gal-7 CRD (PDB 4GAL) (Fig. 6C,D). LNT did so too for Gal-3, as also known from crystallography (PDB 4LBN), and binding of the LNT isomer had been linked to reduction of NT back-folding and enhanced tendency for Gal-3 self-aggregation^56^. Although most of the residues affected are the same as those found for the binding of LacNAc (Fig. 5), additional ones are also perturbed at the base of the S-face β-sheet, most probably due to the increased length of LNT. This was also the case with the binding of KSDS. Contact formation of LNT to Gal-3 also induces some resonance broadening of residues at the CRD S-face binding site (Fig. 6E) due to the exchange process at this LNT:Gal-3 molar ratio. Resonance narrowing for F-face and NT residues (Fig. 6E) likely reflects an increase in protein dynamics within those regions due to allosteric effects and/or to a disruption of transient interactions between the NT and CRD F-face as previously reported^44^. With Gal-7, some minimal resonance broadening occurs throughout the protein (Fig. 6F), suggesting overall changes in dynamics, both at the LNT binding site and at the Gal-7 dimer interface, as we discussed above for KSDS.

Because Gal-3 and -7 resonances shift smoothly as the LNT concentration is increased, we can say that binding occurs in the fast exchange regime on the chemical shift time scale, thus allowing K_D_ values to be determined. For Gal-7, a plot of the average ∆δ vs residue number (insert to Fig. 6F) yields a K_D_ value for LNT binding to Gal-7 of about 500 μM. This comparatively low affinity is consistent with data from calorimetric measurements and frontal affinity chromatography runs with LacNAc di- and trimers^23,33^. Proceeding from these experimental data, we performed docking and MD simulations to visualize interactions.

### MD simulations and binding energetics

**Figure 7:**
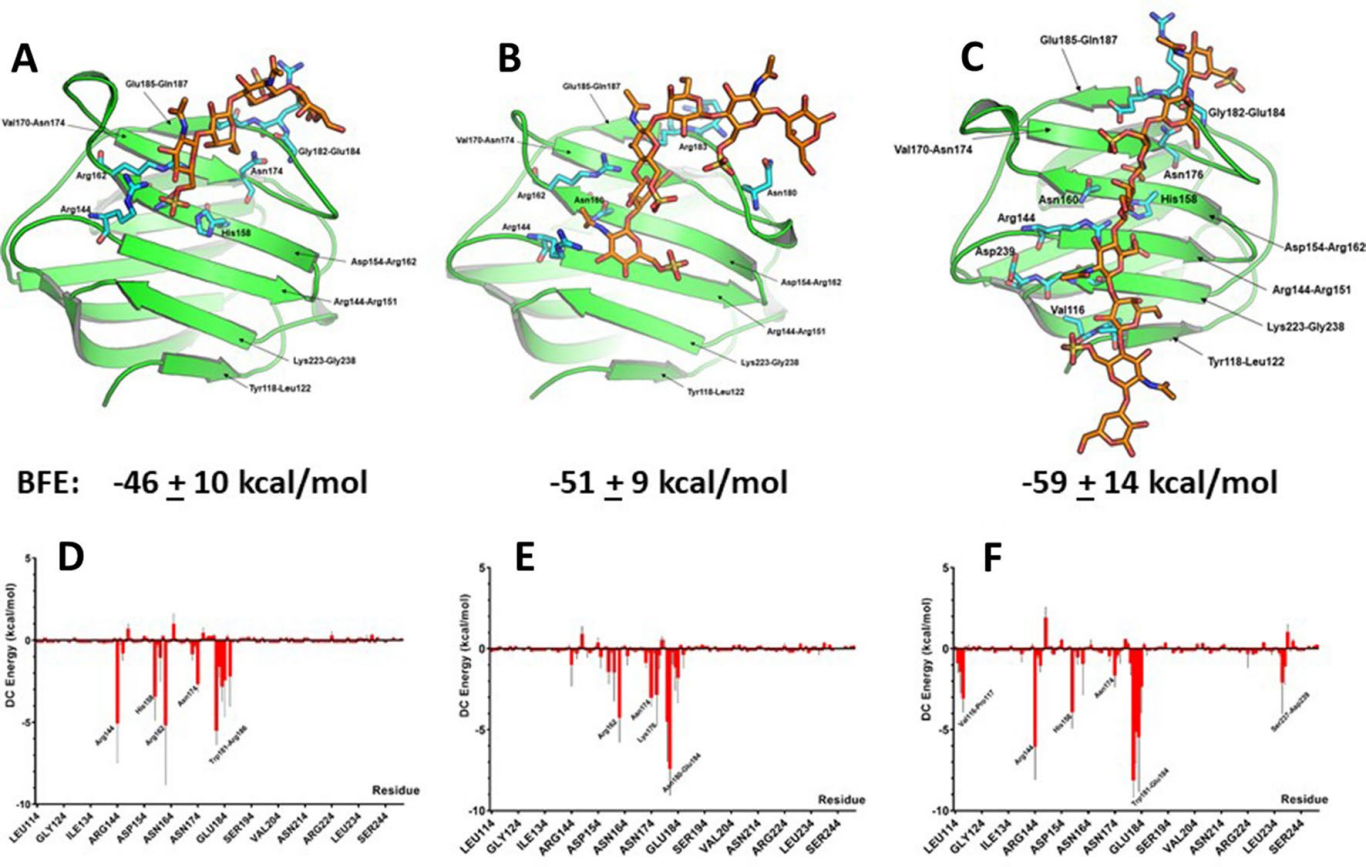
MD simulations and computational calculation of binding energetics for KS-derived oligosaccharides to Gal-3 CRD. Directed by the HSQC-derived chemical shift information, in silico studies were performed by first using the program AutoDock Vina^93^ to dock KS-derived tetra-, hexa- and octasaccharides onto the structure of the Gal-3 CRD (PDB 1A3K), followed by MD simulations and energy minimizations. Energy-minimized structures are shown for the Gal-3 CRD bound to the tetramer (**A**), hexamer (**B**), and octamer (**C**). BFEs are indicated under each structure. Decomposition analysis of free energies of binding (DC) gives, on the per residue basis, free energies for each sugar-loaded structure as shown in (**D**–**F**).

We used our HSQC chemical shift data to guide molecular docking in evidence-based generation of a complex of KS tetra-, hexa- and octasaccharides and the canonical ligand-binding site on the CRD S-face. MD simulations were then run for 50 ns, and Binding Free Energies (BFEs) were calculated. Figure 7A–C show Gal-3-bound oligosaccharide structures after this period of simulation time, with BFEs indicated below each structure. Even though BFEs appear within error to be the same, the trend is such that they become more negative (i.e. binding is stronger) as the size of the KS-derived glycan is increased. The per residue energies shown in Fig. 7D–F indicate that the same set of residues is involved in binding regardless of the size of the oligosaccharide, but that many of the individual per residue energies increase as the length of the oligosaccharide is increased. In addition, in particular the octasaccharide interacts as well with residues within the first strand of the S-face β-sheet, as we observed by HSQC analysis for the binding of the glycosaminoglycan and LNT.

**Figure 8:**
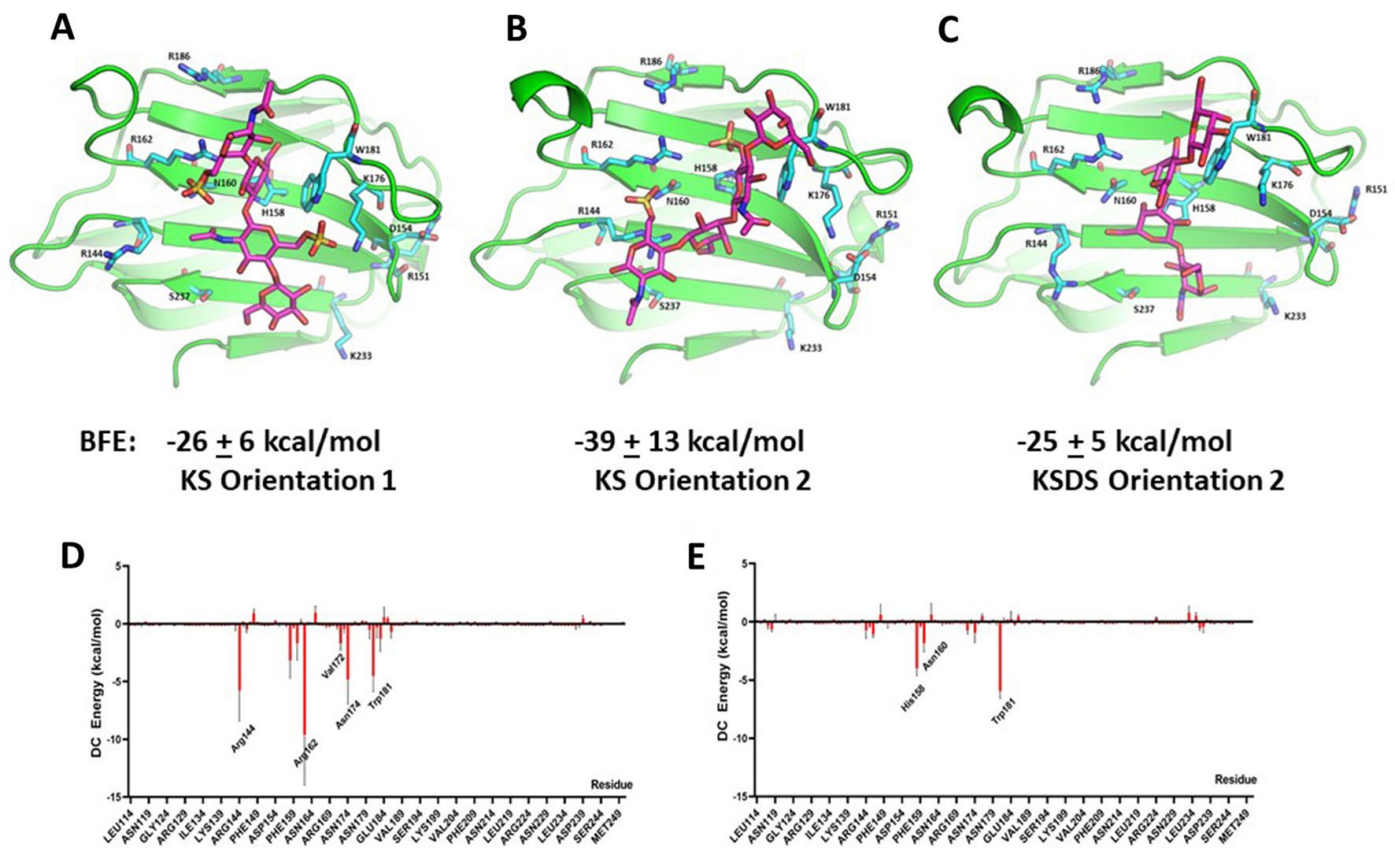
Computational calculation of tetrasaccharides of KS and KSDS binding to Gal-3. In silico studies were performed by using the program AutoDock Vina^93^ to dock KS- and KSDS-derived tetrasaccharides onto the structure of the Gal-3 CRD (PDB 1A3K), followed by MD simulations and energy minimizations. Two different orientations for KS and KSDS tetrasaccharides were used. Orientation 1 placed the ligand with its non-reducing end positioned into the canonical site, whereas orientation 2 (shift by about 180°) brought the reducing end of the glycan into the vicinity of the base of the S-face β-sheet. During MD simulations, orientation 1 for the KSDS tetramer never reached a stable structure during the 50 ns trajectory. Energy-minimized structures are shown for Gal-3 CRD bound to KS with orientation 1 (**A**) and orientation 2 (**B**), as well as for the tetrasaccharide characteristic as structural unit of KSDS in orientation 2 (**C**). BFEs are indicated below each structure. Decomposition analysis of free energies of binding (DC) gave, on the per residue basis, free energies for orientation 2, as shown in (**D**) and (**E**) respectively.

Because negatively charged homogalacturonan poly- and oligosaccharides bind to the Gal-3 CRD with their reducing ends oriented towards the base of the CRD S-face β-sheet (about 180° flipped compared to the β-galactoside lactose)^58^, we wondered whether the chain orientation of KS with its negatively charged sulfate groups (as well as uncharged KSDS) prefer binding to galectins in a similar fashion. To address this issue, we placed KS and KSDS tetrasaccharides in two opposing orientations (1 and 2) within the Gal-3 CRD. In orientation 1, the tetrasaccharide was placed with its non-reducing end positioned as with lactose. In orientation 2, the tetrasaccharide was turned lengthwise by about 180°, such that the reducing end of the tetrasaccharide was in that position. The final KS-loaded structures (orientations 1 and 2) following 50 ns of simulation time are shown in Fig. 8A,B. With the KSDS tetrasaccharide, only orientation 2 remained stable during the simulation (Fig. 8C). In either case, BFEs for orientation 2 were higher than those for orientation 1, with per residue energies for KS and KSDS tetrasaccharide binding in orientation 2 shown in Fig. 8D,E. In this regard, KS tetrasaccharide-bound orientation 2 positions the sulfate groups more favorably for electrostatic interactions with Arg, Lys and His residues. Comparing KS-loaded and -free states of the Gal-3 CRD, RMSD values of residues (all atoms and backbone-only atoms) within some loops vary considerably, closing in around the KS-derived tetrasaccharide (Supplemental Fig. S5A,B). For Gal-3 in contact with the tetrasaccharide from KSDS, RMSD values indicate smaller conformational changes of these loops (Supplemental Fig. S5C,D), similar in magnitude for the binding of lactose to the Gal-1 CRD^57^. Overall, orientation 2 with the reducing end of the saccharide pointing towards the base of the S-face β-sheet presents a novel mode, in which charged groups of the saccharides come into contact with the Gal-3 CRD.

Because our calculations could be biased by regions with high electrostatic potential (i.e. numerous charged residues), we also performed molecular docking in which the negatively charged KS tetrasaccharide was initially placed near a positive-patch (H223-K227, HRVKK) on Gal-3. Although molecular docking showed that the KS tetrasaccharide could fit into a site near this positive patch, the position of the KS tetrasaccharide did not remain stable at this site during MD simulations (Supplemental Fig. S6). By the end of the simulations, KS had moved away from this site towards the canonical site, with BFE values ending up relatively low (around − 14 to − 17 kcal/mol). On the other hand, BFE values for KS binding at the canonical S-Face of Gal-3 are -26 kcal/mol (orientation 1, Fig. 8A) and − 39 kcal/mol (orientation 2, Fig. 8B). Thus, binding of KS (and KSDS) to the S-Face of Gal-3 is not biased by positively charged patches elsewhere on the surface of Gal-3.

Prompted by these findings, as well as by our data on Gal-7 and the known binding parameters for polyLacNAc Gal-1 and -9N, we performed the same MD simulations with the CRDs of Gal-1, Gal-7 and Gal-9N. BFE values for KS and KSDS tetramers in orientations 1 and 2 are given in Table 1. This affinity ranking follows the same trend as that reported by Iwaki et al.^22^ using frontal affinity chromatography. Interestingly, BFEs for Gal-1 in any state are overall the least favorable, whereas those for Gal-9N are close to those for Gal-3 which has the most favorable binding among these galectins. Compared to Gal-3 CRD, moreover, we observed similar RMSD changes (backbone and all atoms) for Gal-9N when comparing KS- (and KSDS-) loaded and free states (Supplemental Fig. S7A–D).

**Table 1:** Binding Free Energies (BFEs, kcal/mol) for KS and KSDS tetramer binding to the CRDs of the given galectins in orientations 1 and 2.

	Orientation 1		Orientation 2	
	KS	KSDS	KS	KSDS
Gal-1	− 13 ± 6	− 7 ± 7	− 21 ± 8	− 16 ± 4
Gal-3	− 26 ± 6	ND	− 39 ± 13	− 25 ± 5
Gal-7	− 22 ± 13	− 16 ± 5	− 25 ± 15	− 19 ± 8
Gal-9N	− 30 ± 7	− 12 ± 7	− 34 ± 13	− 18 ± 7
Gal-9C	− 27 ± 8	− 19 ± 4	− 28 ± 9	− 22 ± 6

## Conclusions

Mapping of KS expression has revealed a wide distribution, prominently in cornea and cartilage, but also in epithelia and the central and peripheral nervous systems^11,16,49,59,60^. Intriguingly, this pattern matches the profile for the expression of Gal-3 (originally termed Mac-2 antigen^61,62^), such that this lectin appears to have been predestined as a receptor for this glycosaminoglycan. Gal-3 joins the list of documented binding partners for corneal KS, such as tyrosine protein kinases (like Ephrin A1 and B1-4), nerve growth factor receptors, semaphorins and other nerve function proteins such as synaptotagmin-2 or Robo-Slit system constituents^60,63^. Adding results from other binding assays^22^ to our present MD-based analysis, Gal-9N also assumes such a status. Our study characterizes the molecular details of the contact, identifies a favorable impact of sulfation, and allows estimation of binding stoichiometry to Gal-3. Thus considering its interactions with *N*- and *O*-glycosylated proteins, such as clusterin or mucins on mucosal surfaces (MUC1/MUC16)^34,64,65^, Gal-3 (and also the hetero-bivalent Gal-9) can act as a molecular glue for the integrity of mucosal barriers. *Mutatis mutandis,* a similar function has been postulated for the eye lens galectin GRIFIN to help organize lens crystallins into their characteristic biological glass-like tight packing^66–68^.

**Figure 9:**
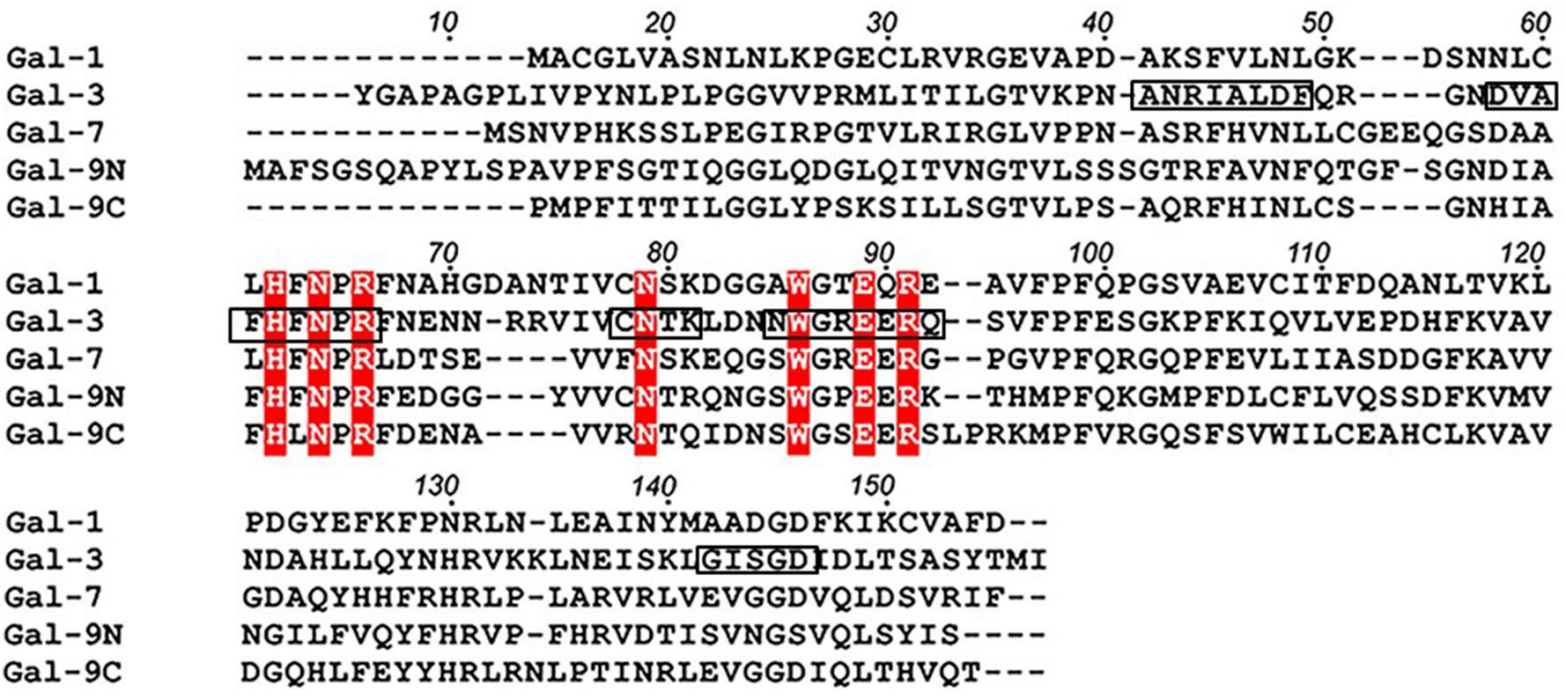
Comparison of amino acid sequences for galectins. The amino acid sequences for Gal-1, -3, -7, and -9N are aligned for comparison. Key residues within the canonical sugar-\binding site are highlighted in red, and segments, for which our data indicate that KS primarily interacts, are identified (boxed in) on the Gal-3 sequence.

In terms of conserved KS binding to the galectins under study, Fig. 9 compares the amino acid sequences for Gal-1, -3, -7, and -9N. Key residues within the canonical sugar-binding site are highlighted in red, and segments for which our data indicate that KS primarily interacts with, are identified (boxed in) on the Gal-3 sequence. One caveat here is that interpretation of our Gal-7 HSQC data is complicated due to the combined effects from KS binding and shifts in the Gal-7 monomer–dimer equilibrium. Nonetheless, KS indeed apparently binds to Gal-7 as with the other galectins. On the glycosaminoglycan side, versatility to act as a molecular glue is further achieved by an α2,3-sialylated branch that is a counter-receptor for Gal-8N (aside from Gal-3)^22^ and for human siglec-8^69^. The 6′-*O*-sulfation of Gal serves as a secondary motif for the binding of human siglec^70^ (although this cannot be extrapolated among species), because murine siglec-F ligands do not require 6′-*O*-sulfation by any of the two galactose 6-*O*-sulfotransferases (KSGal6ST, C6ST-1)^71^. Of note, the increased presence of chondroitinase ABC-sensitive proteoglycans may compensate for the loss of KS in the cornea of mice deficient in β3GnT-7^72^. Fittingly, Gal-3 and -9 share affinity for desulfated chondroitin sulfate^22^, and Gal-3 binds chondroitin sulfates-A, -B and -C, with binding being blocked by lactose^73^.

Considering its abundance in sulfation and positions of sulfate groups, KS can act as a versatile molecular switch for lectin affinity. Whereas galactose 6-*O*-sulfation masks disaccharide binding to galectin, this substitution provides for a high-affinity docking point for C-type lectin langerin^74–76^. Dynamic modulation of sulfation can thus contribute to make the KS platform a multi-purpose binding partner for tissue lectins^77^. The presence/absence of an anionic group is similarly found with glycolipids, where the ganglioside GD1a to GM1 conversion by sialidase transforms a ligand for myelin-associated glycoprotein (siglec-4) and Gal-8 (i.e. GD1a) to a Gal-1, -2, -3 and -7 counter-receptor with functional significance^78–80^. Considering the physiological significance of polyLacNAc stretches (i.e. the backbone of KS) in β1,4(6)-branched N-glycans and core 2/4 *O*-glycans and their importance as galectin counter-receptors^6^, KS is thus a variation of the molecular theme that generates high-affinity docking sites by tandem-repeats of the KS disaccharide unit.

Our report thus highlights the emerging significance for KS as a counter-receptor for distinct galectins, in situ in likely interplay with prominent glycoproteins like aggrecan, CD44, MUC1, phosphocan or podocalyxin. Aside from identifying structural details of the interaction with natural forms of galectins, the availability of variants of the galectin structural architecture (e.g. Gal-1-like homodimers of Gal-3^81^) should allow for delineation of the relevance of topological aspects of CRD presentation for a physiological significance of KS-galectin recognition, a means to contribute to breaking the sugar code by manipulating lectin structure (termed lectinology 4.0^82^). Concerning sulfation heterogeneity, the availability of synthetic homogeneous KS oligo- and polysaccharides is instrumental to study structure–activity relationships of galectins with this pleiotropically active glycosaminoglycan^83,84^. Overall, our structural findings invigorate study of functional aspects of dynamic changes of KS sulfation (called sulfation code), as well as for its presentation by particular proteins as carriers.

## Materials and methods

### Galectin expression and purification

Full-length human Gal-3 (residues 1–250) and its Gal-3 CRD (residues 108–250) were recombinantly produced with [^15^N]NH_4_Cl as medium additive, purified by affinity chromatography on lactosylated Sepharose 4B as a crucial step, and checked for purity and activity as previously described^44^. Uniformly ^15^N-labeled Gal-7 was produced using commercial, isotopically enriched medium with a yield of about 40 mg protein per 100 mL stock solution^53^. Sample characteristics and purity were assessed by using one- and two-dimensional electrophoresis, gel filtration and nano-electrospray ionization mass spectrometry, activity by hemagglutination and proliferation assays^85^.

### Keratan sulfate preparation

Keratan polysaccharide was prepared from bovine corneal keratan sulfate (KS, 5 mg) isolated by the method previously reported^86^. KS is composed of β-(1 → 4)-d-galactose (Gal)-β-(1 → 3)-*N*-acetyl-d-galatosamine (GalNAc) disaccharide repeating units with diversely-linked sulfated groups. KS was converted to its pyridine salt by treatment with Amberlite IR-120 (H^+^ form) followed by filtration and neutralization using pyridine. KS^.^Py salt (6 mg) as white powder was obtained after freeze-drying. Afterwards, solvolytic desulfation^87^ was applied on the pyridine salt of KS as follows. KS^.^Py salt (6 mg) was dissolved in dimethyl sulfoxide (900 μL) containing 10% of water (100 μL), and the reaction solution was stirred at 80 °C for 5 h. Another portion of water (10 mL) was added into the solution which was dialyzed against distilled water for 2 days. The dialysate was lyophilized to give the keratan polysaccharide (4.5 mg) as off-white powder. This species is termed KSDS for Keratan Sulfate De-Sulfated.

Supplemental Figure S2 shows ^1^H NMR spectra of the starting bovine KS polysaccharide and its desulfated form (KSDS). The change in chemical shifts/intensities for protons on C6 indicates removal of sulfate esters on the keratan polysaccharide. PAGE analysis for the starting KS has been reported by Weyers et al.^86^ (see Fig. 2) with the weight averaged molecular weight being given as ~ 10–15 kDa. Because the molecular weight of desulfated KS could not be analyzed using PAGE due to lack of negative charges once desulfated, it was assessed to be intact by its failure to be lost upon dialysis through 8–10 kDa SpectraPor dialysis membrane (Repligen). In this study, we used a KS molecular weight of 14,300 kDa that is based on the mass of the Gal-GalNAc disaccharide unit in KS and the estimated number of these disaccharide units in the polysaccharide. The molecular weight used here for KSDS was estimated to be ~ 9942 based simply on re-calculation of the KS mass upon removal of the sulfates.

### Synthesis of LacNAc

The tested LacNAc derivative was synthesized in five steps starting from glycosyl donor **1**^88^ and glycosyl acceptor **2**. Regioselective benzylidene ring opening of compound **3**^89^ using Et_3_SiH and trifluoroacetic acid (TFA) afforded glycosyl acceptor **2** in 86% yield. Trimethylsilyl trifluoromethanesulfonate (TMSOTf)-mediated glycosylation resulted in formation of the disaccharide **4** (84% yield). In order to afford the fully deprotected LacNAc derivative (shown in Scheme S1), first the NHTCA (trichloroacetamide) group on disaccharide **4** was successfully converted to the naturally occurring NHAc (acetamide) group to afford compound **5** (83% yield). Zemplen reaction conditions removed the acetate group from the galactose moiety in order to obtain compound **6** (94% yield). Ceric ammonium nitrate (CAN) caused oxidative cleavage successfully deprotected the fluorous tag from the anomeric position to afford compound **7** in 87% yield. Pearlman’s catalyst (Pd(OH)_2_/C) was employed to cleave the remaining benzyl groups to achieve the final desired target LacNAc derivative in 83% yield. The general procedure for fluorous solid-phase extraction of our synthesized LacNAc is provided in Supplemental Materials titled: General procedure for fluorous solid-phase extraction (FSPE). LNT was purchased from Carbosynth LDT (Compton, UK).

### NMR spectroscopy

Uniformly ^15^N-labeled Gal-3 (or Gal-7) was dissolved at a concentration of 20 μM in 20 mM potassium phosphate buffer at pH 6.9, made up using a 95% H_2_O/ 5% D_2_O mixture. ^1^H–^15^N HSQC NMR experiments were used to investigate binding of KS or KSDS to Gal-3^39^ and Gal-7^90^. NMR experiments were performed at 30 °C on Bruker AVANCE III 700 MHz or 850 MHz spectrometers equipped with cryogenically-cooled z-gradient triple resonance probes. Chemical shift perturbations were monitored, using the sequence-specific ^1^H and ^15^N resonances assignments for human Gal-3, Gal-3 CRD, and Gal-7. Chemical shifts were internally referenced to DSS (4,4-dimethyl-4-silapentane-1-sulfonic acid). Raw data were converted and processed by using NMRPipe^91^ and were analyzed by using NMRview^92^.

Chemical shifts, δ, were referenced to 4,4-dimethyl-4-silapentane-1-sulfonic acid, and chemical shift differences (∆δ) were calculated as [(∆^1^H)^2^ + (0.25∆^15^N)^2^ ]^1/2^. Intensity changes (∆Intensity) were calculated as (1 − Int_i_/Int_o_), where Int_o_ is the initial resonance intensity and Int_i_ is the intensity upon addition of KS or KSDS or another saccharide. Using this equation, a value of 1 means that that resonance is no longer observable, and 0 means the absence of resonance broadening, with a negative value indicating increased resonance intensity. In chemical shift or resonance broadening maps, the most perturbed resonances are defined as > 2SD above the average or > 1SD above the average. The 50% bound mark was taken from titration curves at 50% bound.

For pulsed field gradient (PFG) NMR self-diffusion measurements, a solution was made up with KS (4 μM) in 20 mM potassium phosphate buffer with ^2^H_2_O at pD 6.7 adjusted by addition of NaO^2^H or ^2^HCl. To this solution, ^15^N/^13^C-labeled Gal-3 (full-length and CRD) dissolved in the same buffer was added at increasing concentrations as molar ratios of [Gal-3]/[KS]. PFG NMR data were acquired at 30 ***◦***C on a Bruker AVANCE 700 spectrometer, essentially as previously described^51^. For unrestricted diffusion of molecules in isotropic solutions, the PFG NMR signal amplitude, *A*, normalized to the signal in the absence of gradient pulses, is related to the diffusion coefficient *D* by Eq. (1):

1 $$A = \exp \, [ - \gamma^{2} g^{2} \Delta^{2} D(\Delta {-}\gamma /3)]$$

where *γ* is the gyromagnetic ratio, *g* is the magnitude of the magnetic field gradient pulse, ∆ is the time between gradient pulses^51^. Here, we set *g* = 1–75 G/cm, ∆ = 34.2 ms, with a longitudinal eddy-current delay of 100 ms. Each *D* value was determined from a series of 14 1D PFG NMR spectra (256 scans each) using different *g* values. To derive *D* values, the gradient-induced change in amplitude of KS resonances at 3.64 and 3.7 ppm was used. In this spectral region, KS resonance intensity was considerably greater than that from Gal-3. In order to decrease Gal-3 resonance intensity further, ^13^CH and ^15^NH resonances from labeled Gal-3 were not decoupled.

### Molecular dynamics simulations

Similar approaches and protocols were used as previously reported^93^. Briefly, the Amber 14SB force field was applied for proteins, and GLYCAM06 was used for oligosaccharides. KS and KSDS oligosaccharides were initially dock on the galectin structure using the program AutoDock Vina^94^. Complexes were solvated by TIP3P water models with a box size of 10 Å and were then subjected to energy minimization (5,000 steps of steepest descent followed by 5,000 steps of conjugate gradient algorithm) in order to optimize the complexes and remove close contacts. Subsequently, a position-restrained phase of MD simulations was carried out for 500 ps by first slowly heating up the systems from 0 to 300 K for 100 ps and then maintaining the temperature at 300 K for another 400 ps. During this phase, a soft-force constraint (10 kcal/mol Å^2^) was applied to restrain the complexes. Finally, free MD simulations were performed for 50 ns.

Essential parameters (e.g. temperature (300 K), pressure (1 bar) and time steps (2 fs with SHAKE constraint)) were set at standard values as indicated during the free MD simulations. Energy minimization and MD simulations were employed by using AMBER16 program. BFE calculations were performed by extracting trajectories during the 30–50 ns period of MD simulations (100 snapshots), and the molecular mechanics/ generalized Born surface area (MM/GBSA) approach (generalized Born model 5 with standard parameters) was used for this purpose. The 3D structures of galectins were obtained from the Protein Data Bank: Gal-1 (PDB code 1W6N), Gal-3 (PDB code 1A3K), Gal-7 (PDB code 5H9S), Gal-9C (PDB code 3NV3), and Gal-9N (PDB code 2EAL). The Uni-Prot IDs used for sequence alignment by the “clustal Omega” program of the UniProtKB are P09382 (Gal-1), P17931 (Gal-3), P47929 (Gal-7), O00182 (Gal-9). In some instances, the program Glycotech Vina^95^ that is optimized for sulfated glycans was employed, and results followed the same trends as with the program AutoDock Vina^94^. Using Glycotech Vina, derived binding free energies were relatively low (− 6 kcal/mol for orientation 1 and − 9 kcal/mol for orientation 2) compared to those from use of AutoDock Vina (− 26 kcal/mol for orientation 1 and − 39 kcal/mol for orientation 2).

In all simulations, we used a pH value ~ pH 7 where histidine can have three different protonation states: protonation at the NE atom (HIE), at the ND atom (HID), and a double protonation at both NE and ND atoms (HIP). His158, for example, is located at the CRD binding site, and its protonation state could influence binding to KS and KSDS. However, because we focused this study on relative binding free energies, we set the His protonation state to the same value (HIE at pH 7 by default), and all systems remained stable during the simulations.

## Supplementary information

Supplementary file1

Supplementary file2

Supplementary file3

Supplementary file4

Supplementary file5

Supplementary file6

Supplementary file7

Supplementary file8

Supplementary file9

Supplementary file10

## Abbreviations

BFE: Binding free energy
CRD: Carbohydrate recognition domain
Gal: Galactose
Gal-1: Galectin-1
Gal-3: Galectin-3
LNT: Lacto-*N*-tetraose
NT: N-terminal tail
Gal-3 FL: Full-length Gal-3 (CRD + NT)
Gal-3 CRD: Truncated (tr)Gal-3
Gal-7: Galectin-7
GlcNAc: *N*-Acetylglucosamine
Gal-9: Galectin-9
HSQC: Heteronuclear single quantum coherence
KS: Keratan sulfate
KSDS: Desulfated KS
MD: Molecular dynamics
NMR: Nuclear magnetic resonance
