# Supplementary figures and images for: Structural insight into the binding of human galectins to corneal keratan sulfate, its desulfated form and related saccharides
Source: Sci Rep. 2020 Sep 24;10:15708. doi: 10.1038/s41598-020-72645-9 (PMC7515912; doi:10.1038/s41598-020-72645-9)

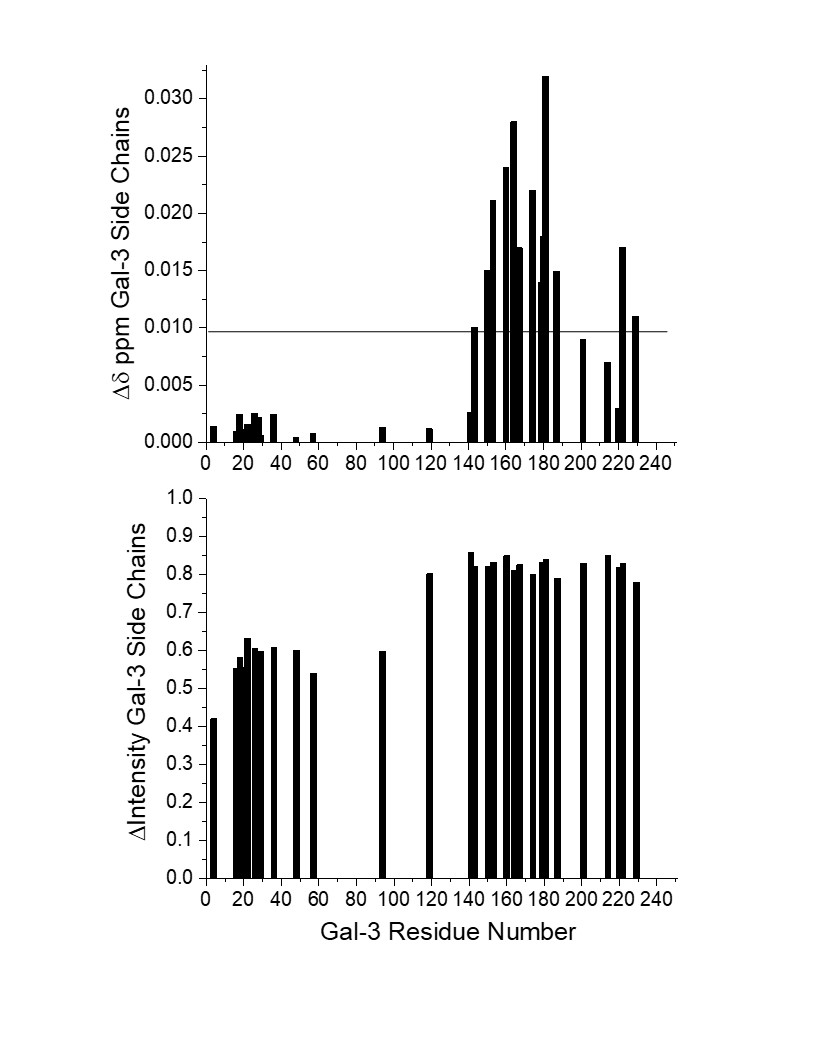

Supplement: Supplementary file 1 — Supplementary file1 [file 41598_2020_72645_MOESM1_ESM.jpg]

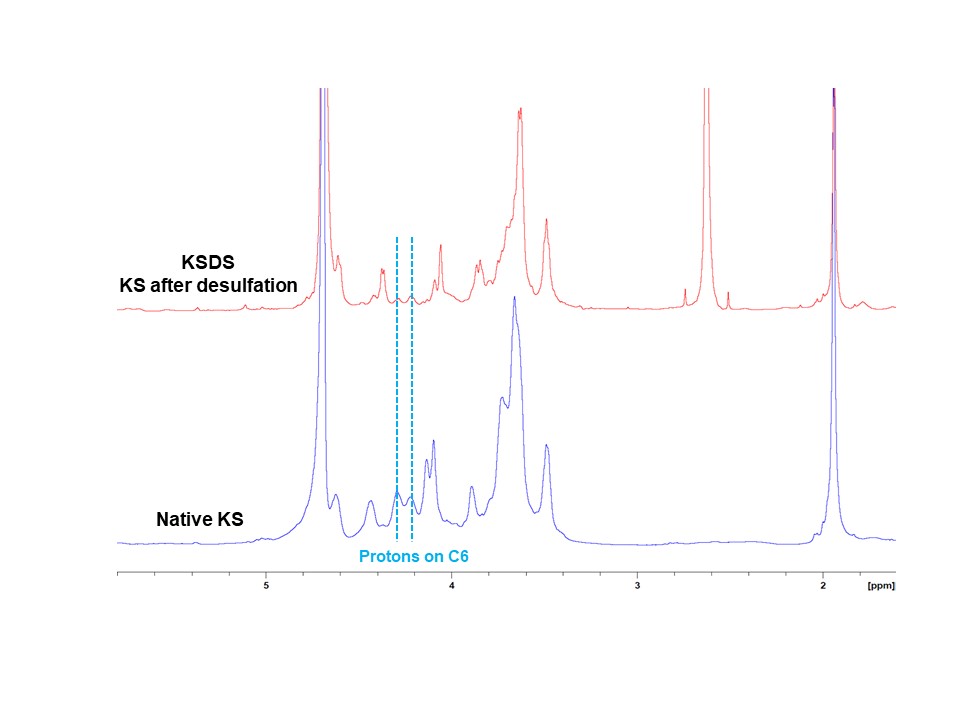

Supplement: Supplementary file 2 — Supplementary file2 [file 41598_2020_72645_MOESM2_ESM.jpg]

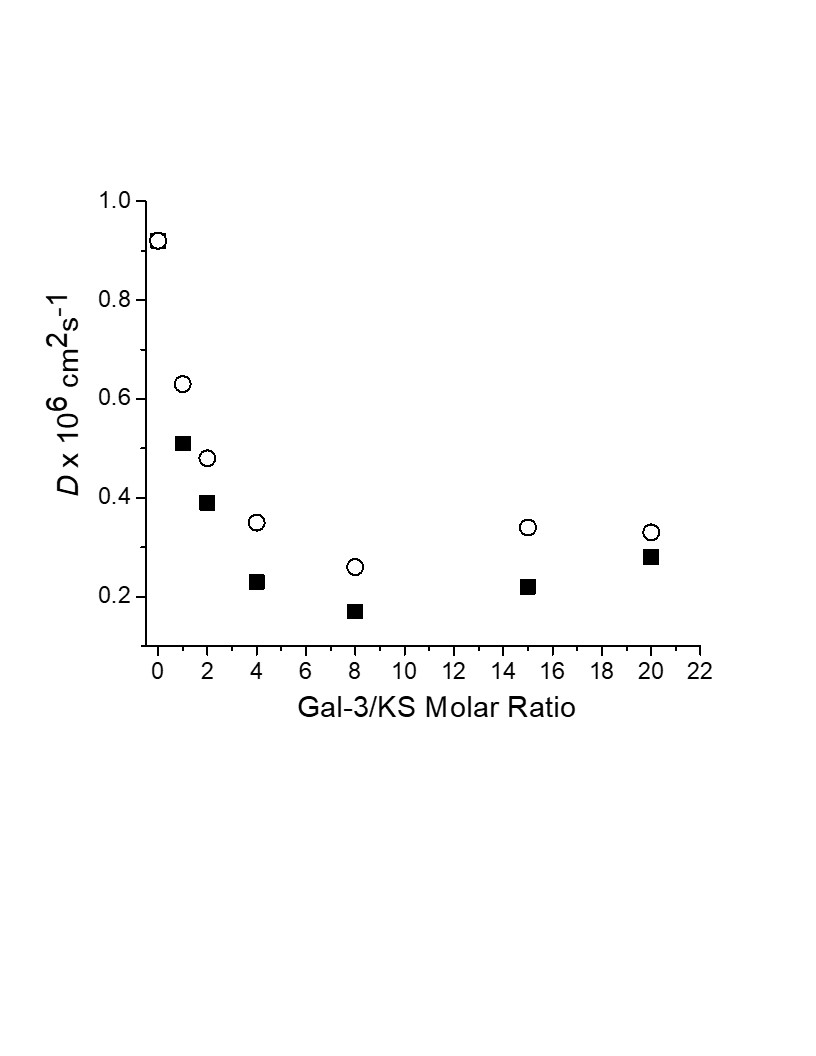

Supplement: Supplementary file 3 — Supplementary file3 [file 41598_2020_72645_MOESM3_ESM.jpg]

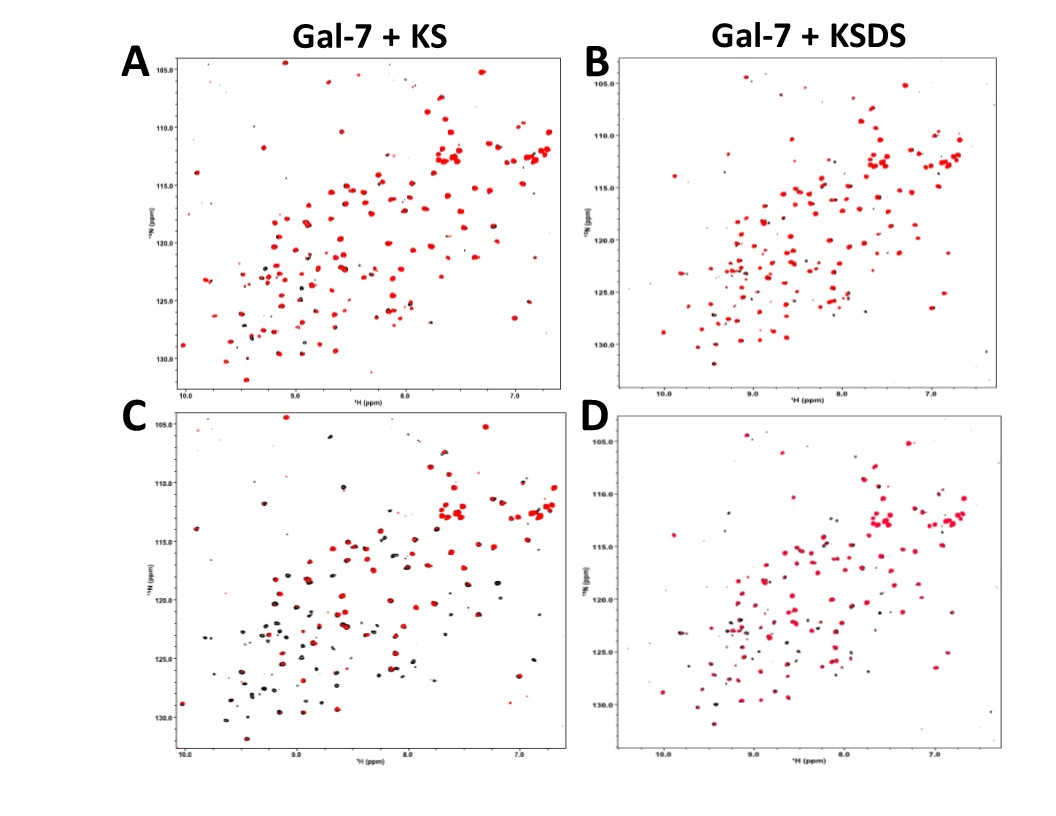

Supplement: Supplementary file 4 — Supplementary file4 [file 41598_2020_72645_MOESM4_ESM.jpg]

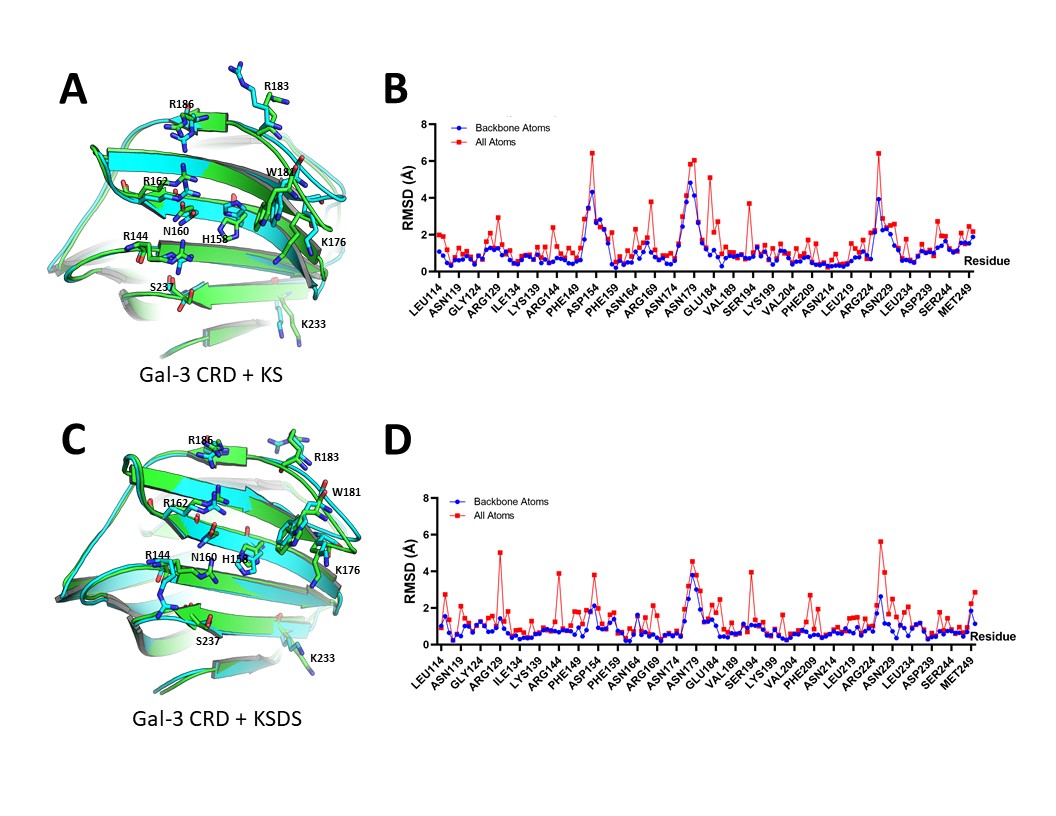

Supplement: Supplementary file 5 — Supplementary file5 [file 41598_2020_72645_MOESM5_ESM.jpg]

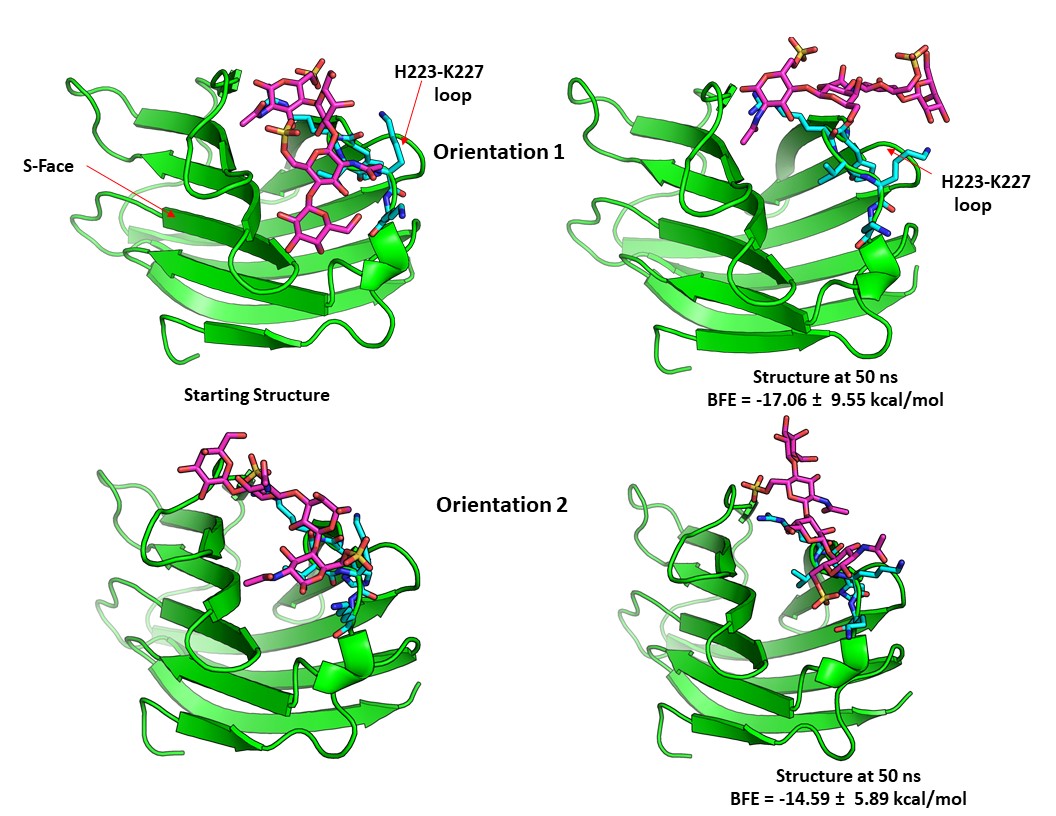

Supplement: Supplementary file 6 — Supplementary file6 [file 41598_2020_72645_MOESM6_ESM.jpg]

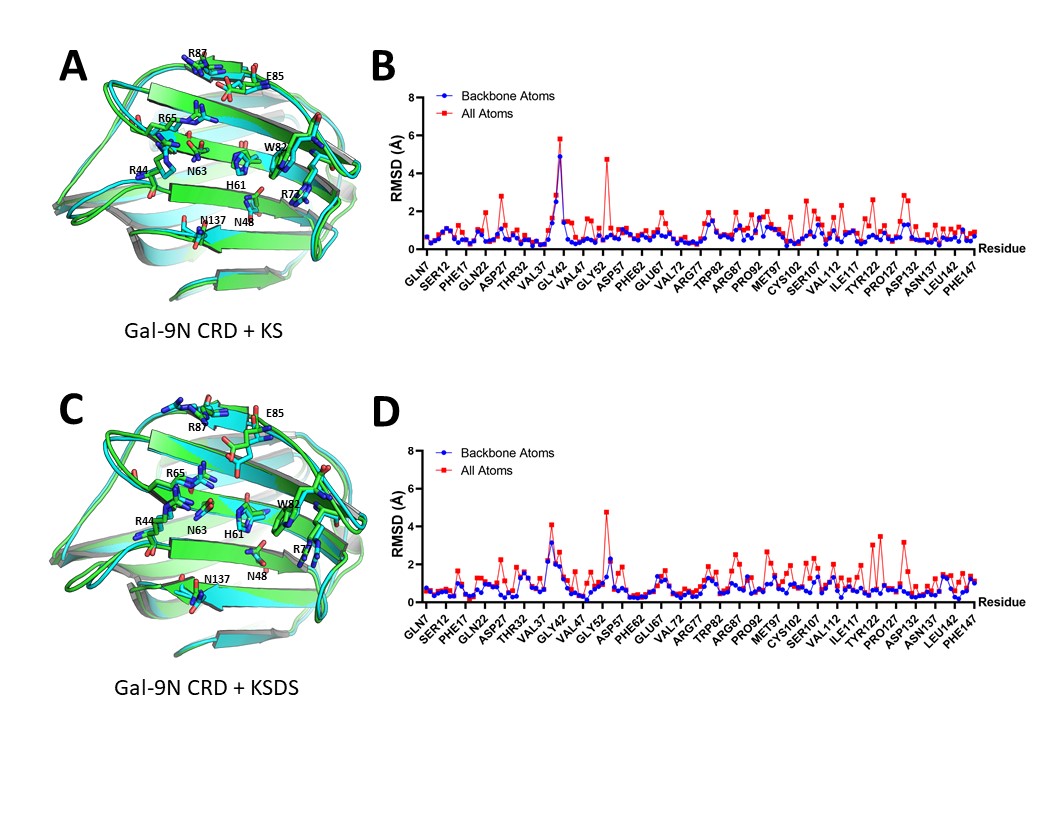

Supplement: Supplementary file 7 — Supplementary file7 [file 41598_2020_72645_MOESM7_ESM.jpg]
