# Supplementary material for: Structural insight into the binding of human galectins to corneal keratan sulfate, its desulfated form and related saccharides
Source: Sci Rep. 2020 Sep 24;10:15708. doi: 10.1038/s41598-020-72645-9 (PMC7515912; doi:10.1038/s41598-020-72645-9)
Supplement: Supplementary file 8 — Supplementary file8 [file 41598_2020_72645_MOESM8_ESM.docx]

**Scheme S1:** Successful synthesis of LacNAc derivative.

**Reagents and conditions:** (a) Et_3_SiH, TFA, CH_2_Cl_2_, 0 °C to 25 °C, 4 h, 86%; (b) TMSOTf, CH_2_Cl_2_, 0 °C, 5 min, 84%; (c) Bu_3_SnH, AIBN, Benzene:DMAC (4:1 v/v), 25 °C to 80 °C, 4 h, 83%; (d) Na, MeOH, 25 °C, 3 h, 94%; (e) CAN, CH_3_CN:H_2_O (4:1), 0 °C to 25 °C, 0.5 h, 87%; (f) H_2_/Pd(OH)_2_-C, MeOH, 25 °C, 24 h, 83%.
