# Supplementary material for: Structural insight into the binding of human galectins to corneal keratan sulfate, its desulfated form and related saccharides
Source: Sci Rep. 2020 Sep 24;10:15708. doi: 10.1038/s41598-020-72645-9 (PMC7515912; doi:10.1038/s41598-020-72645-9)
Supplement: Supplementary file 9 — Supplementary file9 [file 41598_2020_72645_MOESM9_ESM.docx]

**Supplemental Method for Sugar Synthesis**

**General procedure for Fluorous Solid-Phase Extraction (FSPE)**

The crude reaction mixture (less than 0.3 g) was dissolved in DMF (0.4 mL) and loaded onto the fluoroflash silica gel cartridge. Non-fluorous compounds were eluted with 10 mL 80% methanol (MeOH) in water and fluorous compounds were eluted with 10 mL MeOH. The fluorous fraction was concentrated to obtain the desired product.

**2-(4-((4,4,5,5,6,6,7,7,8,8,9,9,10,10,11,11,11-heptadecafluoroundecyl)oxy)phenoxy)ethyl 3,6-di-*O*-benzyl-2-deoxy-2-trichloroacetamido-*β*-D-glucopyranoside (2):** To a solution of compound **3** (0.21 g, 0.19 mmol) in anhydrous dichloromethane (10 mL) were added triethylsilane (0.180 mL, 1.15 mmol) and TFA (trifluoroacetic acid) (0.060 mL, 0.76 mmol) at 0 °C under argon. The reaction mixture was then allowed to warm to room temperature (25 °C) and continued to stir for 4 h at the same temperature until the starting material was completely consumed by think layer chromatography (TLC). The reaction was quenched by addition of triethylamine drop wise until the reaction mixture was neutral to a litmus paper and diluted with dichloromethane (20 mL). The organic layer was washed with water (3 X 20 mL), saturated solution of sodium bicarbonate (3 X 20 mL) and brine solution (2 X 20 mL). The organic phase was dried over Na_2_SO_4_ and the solvent was removed *in vacuo****.*** The resulting crude material was purified via FSPE to afford compound **2** (0.18 g, 0.16 mmol, 86 %) as a white solid. **^1^H NMR** (400 MHz, CDCl_3_) δ 7.44 – 7.19 (m, 10H), 6.92 (d, *J* = 7.5 Hz, 1H), 6.79 (s, 4H), 5.02 (d, *J* = 8.3 Hz, 1H), 4.84 – 4.70 (m, 2H), 4.58 (q, *J* = 12.0 Hz, 2H), 4.13 – 3.87 (m, 7H), 3.81 – 3.67 (m, 3H), 3.61 – 3.43 (m, 2H), 2.73 (s, 1H), 2.30 (m, 2H), 2.06 (m, 2H). **^13^C NMR** (101 MHz, CDCl_3_) δ 161.9, 153.0, 152.9, 138.0, 137.5, 128.6, 128.5, 128.1, 128.0, 127.9, 127.8, 115.5, 115.4, 99.5, 92.4, 79.7, 74.8, 73.8, 73.4, 70.4, 68.1, 67.8, 66.9, 58.3, 29.7, 27.9, 20.6. **HRMS-ESI-TOF (m/z):** [M+Na]^+^ Calculated for C_41_H_37_Cl_3_F_17_NO_8_ **1124.1193,** Found **1124.1188.**

**2-(4-((4,4,5,5,6,6,7,7,8,8,9,9,10,10,11,11,11-heptadecafluoroundecyl)oxy)phenoxy)ethyl (2-*O*-acetyl-3,4,6-tri-*O*-benzyl- *β*-D-galactopyranosyl)-(1→4)-3,6-di-*O*-benzyl-2-deoxy-2-trichloroacetamido-*β*-D-glucopyranoside (4):** To a vigorously stirred solution of glycosyl donor **1** (43.0 mg, 68.0 µmol) and glycosyl acceptor **2** (0.050 g, 0.045 mmol) in anhydrous dichloromethane (1.0 mL) under argon, catalytic TMSOTf (trimethylsilyl trifluoromethanesulfonate) (1.23 µL, 6.75 µmol) was added at 0 °C and the reaction mixture was stirred for 5 min at the same temperature until the acceptor was completely consumed shown by TLC. The reaction mixture quenched by the addition of triethylamine drop wise until the reaction mixture was neutral to a litmus paper. The reaction mixture was then diluted with dichloromethane (3 mL) and the solvent was removed under reduced pressure. The resulting crude material was purified via FSPE to afford compound **4** (58.0 mg, 37.0 µmol, 84 %) as white foam. **^1^H NMR** (400 MHz, CDCl_3_) δ 7.40 – 6.98 (m, 27H), 6.79 (d, *J* = 4.2 Hz, 4H), 5.36 – 5.25 (m, 1H), 4.99 – 4.81 (m, 3H), 4.69 – 4.22 (m, 9H), 4.16 – 3.81 (m, 9H), 3.78 – 3.64 (m, 3H), 3.60 – 3.45 (m, 2H), 3.36 (m, 3H), 2.29 (m, 2H), 2.11 – 2.01 (m, 2H), 1.98 (s, 3H). **^13^C NMR** (101 MHz, CDCl_3_) δ 169.4, 161.8, 153.1, 152.9, 138.5, 138.2, 137.9, 137.8, 128.4, 128.4, 128.4, 128.2, 128.1, 128.1, 128.0, 127.9, 127.8, 127.7, 127.5, 127.4, 127.3, 115.6, 115.4, 100.7, 99.7, 92.4, 80.1, 76.0, 75.4, 74.7, 74.3, 73.5, 73.4, 73.3, 72.6, 71.9, 71.8, 68.4, 67.9, 67.8, 66.9, 56.4, 29.7, 28.2, 27.9, 27.7, 21.0, 20.6, 20.6. **HRMS-ESI-TOF (m/z):** [M+Na]^+^ Calculated for C_70_H_67_Cl_3_F_17_NO_14_ **1598.3248,** Found **1598.3209.**

**2-(4-((4,4,5,5,6,6,7,7,8,8,9,9,10,10,11,11,11-heptadecafluoroundecyl)oxy)phenoxy)ethyl (2-*O*-acetyl-3,4,6-tri-*O*-benzyl- *β*-D-galactopyranosyl)-(1→4)-2-acetamido-3,6-di-*O*-benzyl-2-deoxy-*β*-D-glucopyranoside (5):** To a solution of the compound **4** (0.040 g, 0.025 mmol) in benzene (4 mL) and dimethylacetamide (DMAC, 1 mL, 4:1, v/v) were added AIBN (azobisisobutyronitrile) (0.002 g, 0.012 mmol) and tributyltin hydride (0.067 mL, 0.25 mmol), degassed with the flow of argon and stirred at room temperature for 30 min. The reaction mixture was then heated at 80 °C for 4 h. TLC showed complete conversion of the starting material. The reaction mixture was then concentrated. The resulting crude solid material was purified via FSPE. The fluorous fraction was concentrated to afford desired compound **5** (0.03 g, 0.02 mmol, 83%) as a white solid. **^1^H NMR** (500 MHz, CDCl_3_) δ 7.38 – 7.12 (m, 26H), 6.86 – 6.74 (m, 4H), 6.16 (d, *J* = 9.0 Hz, 1H), 5.35 – 5.27 (m, 1H), 4.92 (d, *J* = 11.5 Hz, 1H), 4.74 – 4.32 (m, 11H), 4.12 – 3.90 (m, 9H), 3.84 – 3.70 (m, 5H), 3.57 (d, *J* = 8.2 Hz, 1H), 3.46 (m, 2H), 2.29 (m, 2H), 1.96 (d, *J* = 52.9 Hz, 8H). **^13^C NMR** (126 MHz, CDCl_3_) δ 170.2, 170.2, 153.3, 152.9, 138.5, 138.4, 138.2, 137.9, 137.8, 128.5, 128.4, 128.3, 128.2, 128.1, 127.9, 127.8, 127.8, 127.7, 127.6, 127.4, 127.3, 115.7, 115.4, 100.5, 99.9, 79.8, 76.4, 74.6, 74.4, 74.1, 73.6, 73.6, 73.4, 72.6, 72.5, 72.1, 71.8, 69.7, 68.1, 67.8, 67.6, 66.9, 50.4, 29.7, 28.0, 27.8, 23.2, 21.0, 20.6. **HRMS-ESI-TOF (m/z):** [M+Na]^+^ Calculated for C_70_H_70_F_17_NO_14_ **1494.4423,** Found **1494.4467.**

.

**2-(4-((4,4,5,5,6,6,7,7,8,8,9,9,10,10,11,11,11-heptadecafluoroundecyl)oxy)phenoxy)ethyl(3,4,6-tri-*O*-benzyl-*β*-D-galactopyranosyl)-(1→4)-2-acetamido-3,6-di-*O*-benzyl-2-deoxy-*β*-D-glucopyranoside (6):** To a solution of compound **5** (27.0 mg, 18.0 µmol) in methanol (3.0 mL) was added a pinch of solid sodium metal at ambient temperature under argon. The reaction mixture was stirred for 3 h at ambient temperature and quenched by addition Dowex® (50W X8, 200-400 mesh) (H^+^) cation exchange resin. The resin was filtered over a pad of Celite®, rinsed with methanol. The methanol was removed in vacuo and the trace of methanol or water was removed by co-evaporation with toluene (3x5 mL). The crude white solid product **6** (24.0 mg, 17.0 µmol) was used directly in the next step. **^1^H NMR** (400 MHz, CDCl_3_) δ 7.28 (m, 26H), 6.80 (d, *J* = 3.9 Hz, 4H), 5.66 (d, *J* = 7.7 Hz, 1H), 4.96 – 4.80 (m, 3H), 4.74 – 4.43 (m, 7H), 4.30 (q, *J* = 11.6 Hz, 2H), 4.13 – 3.71 (m, 13H), 3.67 – 3.44 (m, 3H), 3.40 – 3.25 (m, 3H), 2.98 (s, 1H), 2.29 (dd, *J* = 17.6, 8.8 Hz, 2H), 2.05 (d, *J* = 5.8 Hz, 2H), 1.74 (s, 3H). **^13^C NMR** (101 MHz, CDCl_3_) δ 170.4, 153.2, 152.9, 138.9, 138.7, 138.1, 137.9, 128.5, 128.4, 128.3, 128.2, 128.1, 127.8, 127.7, 127.7, 127.6, 127.4, 127.3, 115.6, 115.4, 103.2, 100.3, 81.9, 78.7, 77.3, 77.0, 76.7, 74.6, 74.6, 73.8, 73.5, 73.4, 73.4, 72.8, 72.3, 72.0, 68.7, 68.2, 67.9, 67.8, 66.9, 55.9, 31.9, 29.7, 29.4, 27.9, 22.7, 20.6. **HRMS-ESI-TOF (m/z):** [M+Na]^+^ Calculated for C_68_H_68_F_17_NO_13_ **1452.4316,** Found **1452.4325.**

**1-Hydroxyethyl (3,4,6-tri-*O*-benzyl-*β*-D-galactopyranosyl)-(1→4)-2-acetamido-3,6-di-*O*-benzyl-2-deoxy-*β*-D-glucopyranoside (7):** The compound **6** (24.0 mg, 17.0 µmol) was dissolved in a mixture of acetonitrile and water (0.4 mL, 4:1 acetonitrile/water) and the solution was stirred at 0 °C in an ice bath. Solid ceric ammonium nitrate (28.0 mg, 51.0 µmol) was added portion wise to the reaction mixture at the same temperature. The reaction mixture was then allowed to warm to ambient temperature and stirred for 0.5 h. The reaction was diluted with water (10 mL) and extracted with ethyl acetate several times. The organic layer was dried over Na_2_SO_4_. The solvent was removed *in vacuo* and the resulting crude oil was purified by flash column chromatography on silica gel (5% to 10% methanol gradient in methylene chloride) to afford compound **7** (12.0 mg, 14.0 µmol, 87%) as pale yellow oil. **^1^H NMR** (500 MHz, CDCl_3_) δ 7.36 – 7.03 (m, 25H), 5.59 (d, *J* = 7.5 Hz, 1H), 4.86 – 4.16 (m, 12H), 3.94 – 3.16 (m, 17H), 2.76 (s, 1H), 1.75 (s, 3H). **^13^C NMR** (126 MHz, CDCl_3_) δ 169.5, 137.6, 137.0, 136.8, 127.5, 127.4, 127.3, 127.2, 127.1, 126.9, 126.8, 126.7, 126.6, 126.5, 126.4, 126.4, 102.1, 99.9, 80.8, 77.8, 76.2, 75.9, 75.7, 73.6, 73.5, 72.8, 72.4, 72.4, 71.7, 71.2, 70.8, 67.7, 67.2, 61.0, 54.9, 22.5. **HRMS-ESI-TOF (m/z):** [M+Na]^+^ Calculated for C_51_H_59_NO_12_ **900.3935,** Found **900.3898.**

**1-Hydroxyethyl(*β*-D-galactopyranosyl)-(1→4)-2-acetamido-2-deoxy-*β*-D-glucopyranoside (LacNAc derivative):** Pd(OH)_2_/C (25.0 mg) was added to a solution of the compound **7** (12.0 mg, 14.0 µmol) in methanol (3.0 mL). The reaction mixture was degassed 3 times with argon and stirred for 24 h under an atmosphere of hydrogen. It was then filtered over Celite and washed with methanol (3 X 10 mL). The solution was concentrated to afford the target LacNAc derivative (4.80 mg, 11.0 µmol, 83%) as a white solid. **^1^H NMR** (500 MHz, D_2_O) δ 4.43 (d, *J* = 8.2 Hz, 1H), 4.33 (d, *J* = 7.8 Hz, 1H), 3.88 – 3.76 (m, 4H), 3.72 – 3.50 (m, 16H), 3.49 – 3.36 (m, 3H), 3.20 (s, 1H), 1.89 (s, 3H). **^13^C NMR** (126 MHz, D_2_O) δ 174.7, 102.8, 101.1, 78.4, 75.3, 74.7, 72.5, 72.4, 71.1, 70.9, 68.5, 60.9, 60.5, 55.0, 48.8, 22.1. **HRMS-ESI-TOF (m/z):** [M+Na]^+^ Calculated for C_16_H_29_NO_12_ **450.1588,** Found **450.1594.**

**^1^H NMR, ^13^C NMR and Mass Spectrometry data**

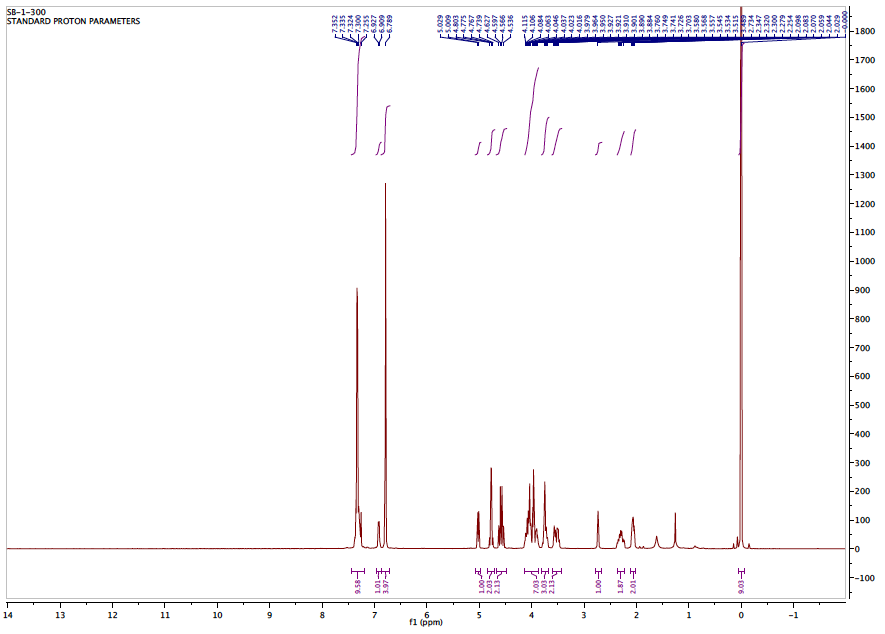


**^1^H NMR** (400 MHz) spectrum of compound **2** in CDCl_3_

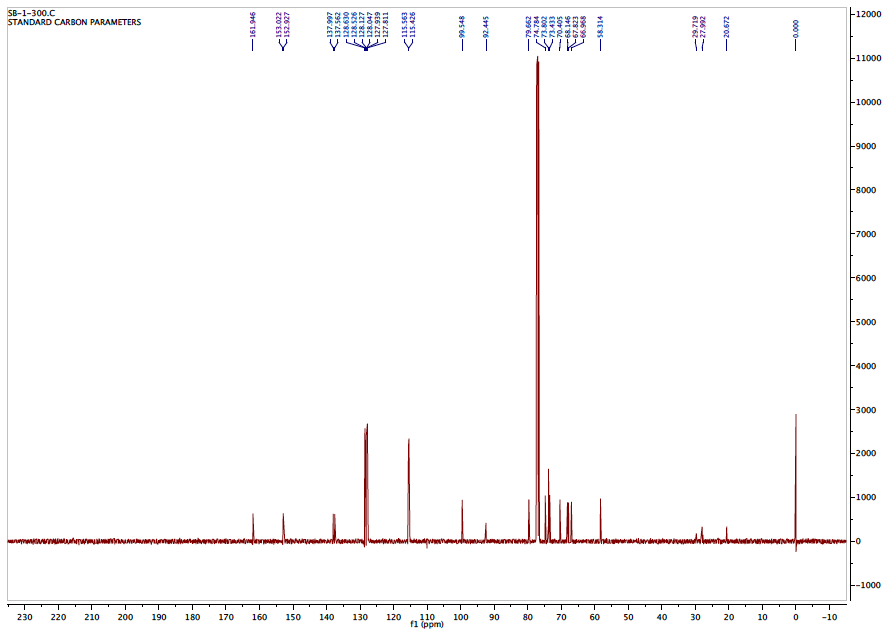


**^13^C NMR** (101 MHz) spectrum of compound **2** in CDCl_3_

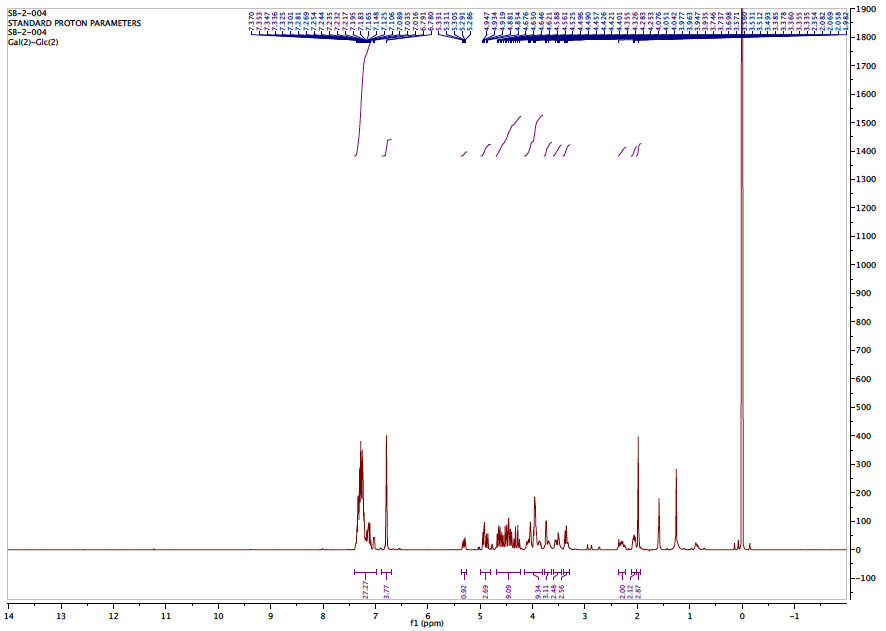


**^1^H NMR** (400 MHz) spectrum of compound **4** in CDCl_3_

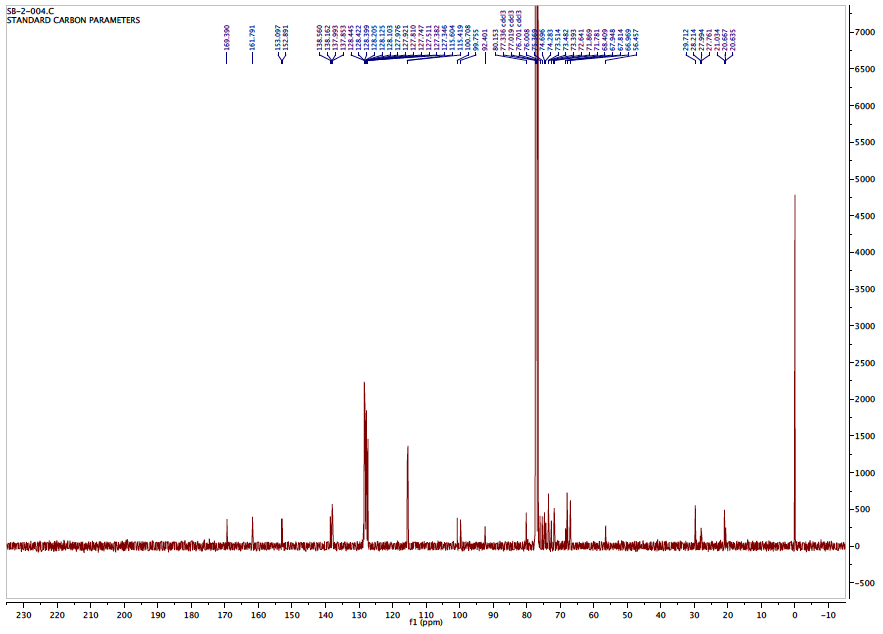


**^13^C NMR** (101 MHz) spectrum of compound **4** in CDCl_3_

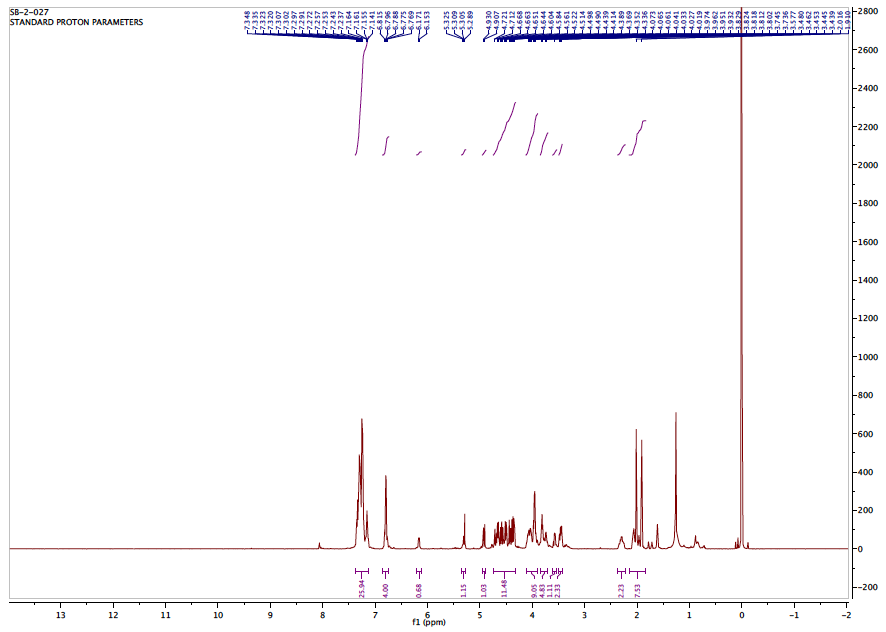


**^1^H NMR** (500 MHz) spectrum of compound **5** in CDCl_3_

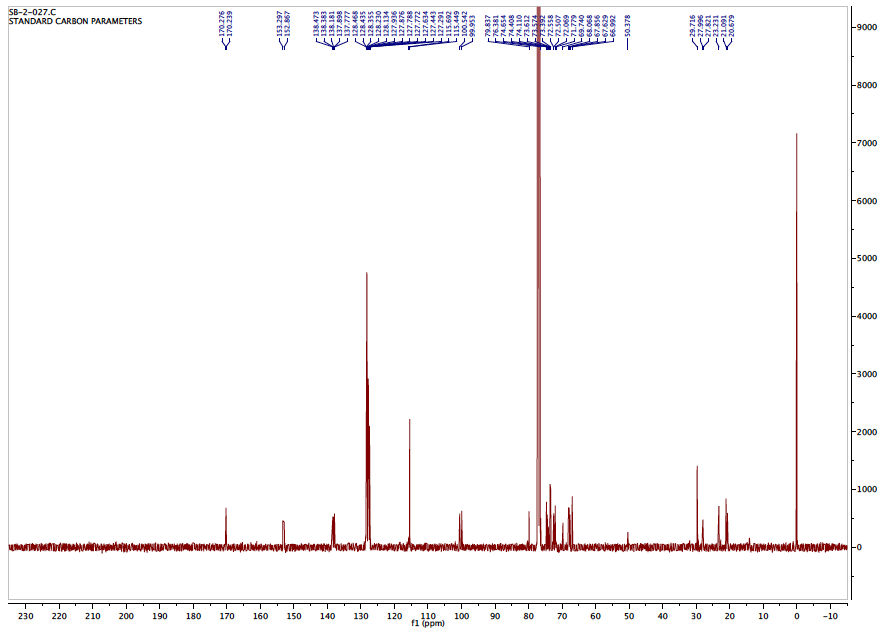


**^13^C NMR** (126 MHz) spectrum of compound **5** in CDCl_3_

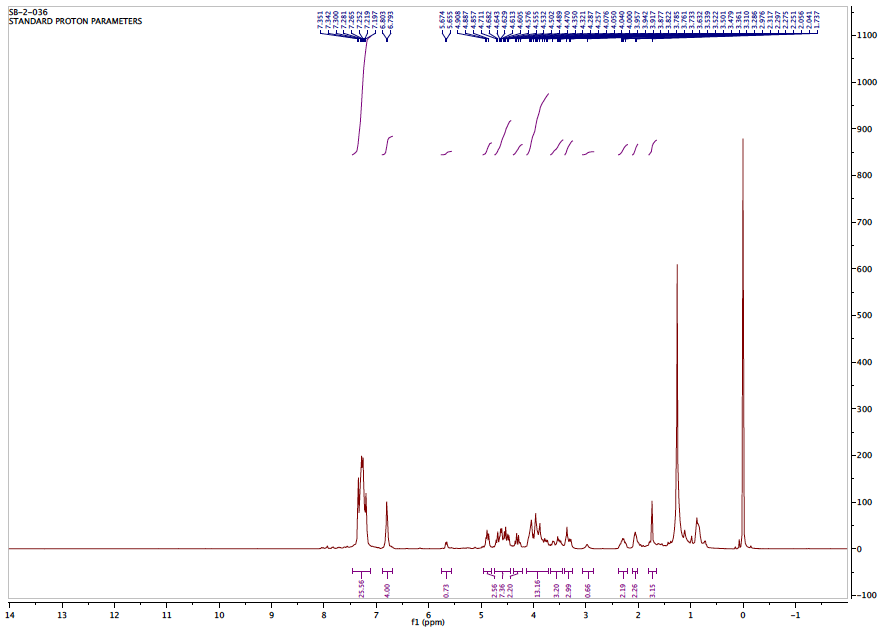


**^1^H NMR** (400 MHz) spectrum of compound **6** in CDCl_3_


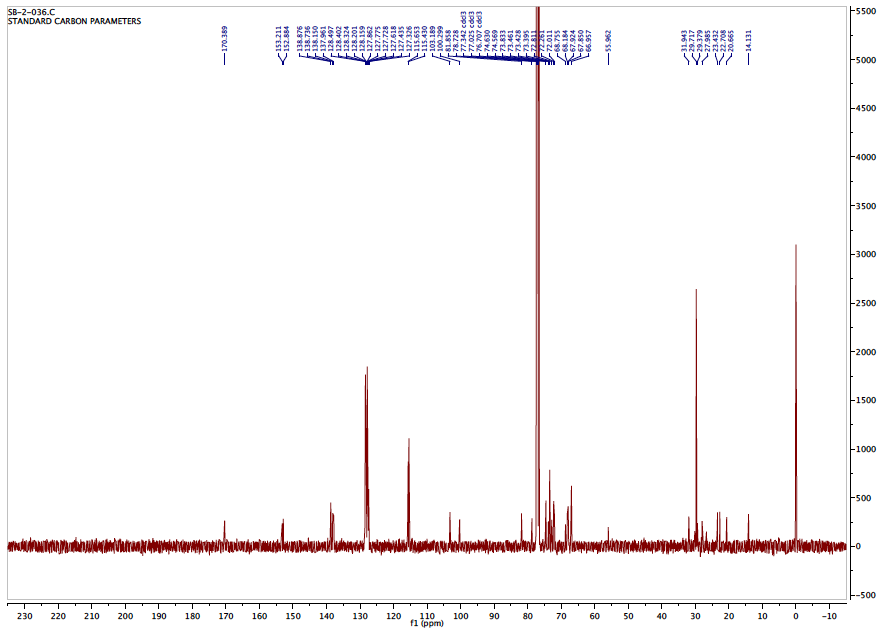

**^13^C NMR** (101 MHz) spectrum of compound **6** in CDCl_3_

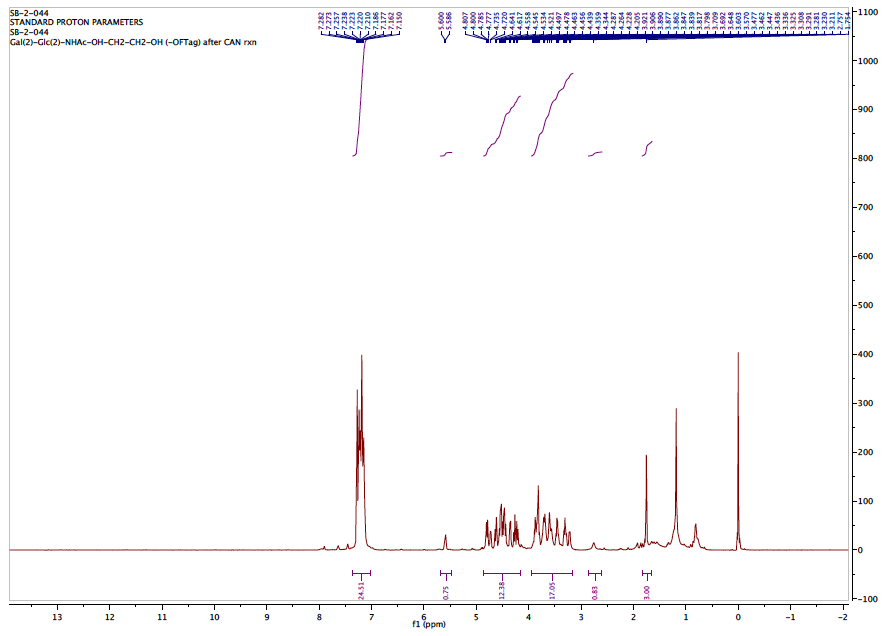


**^1^H NMR** (500 MHz) spectrum of compound **7** in CDCl_3_

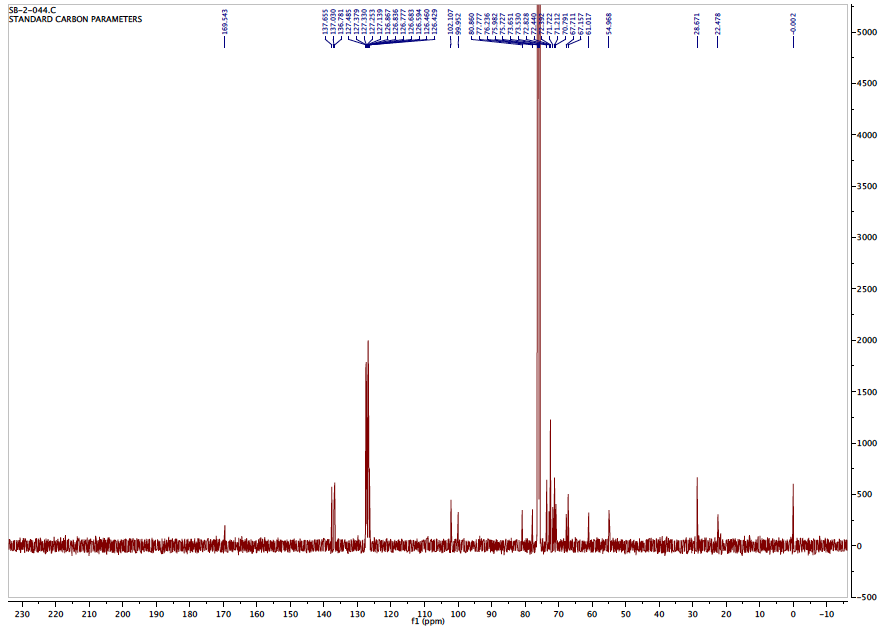


**^13^C NMR** (126 MHz) spectrum of compound **7** in CDCl_3_

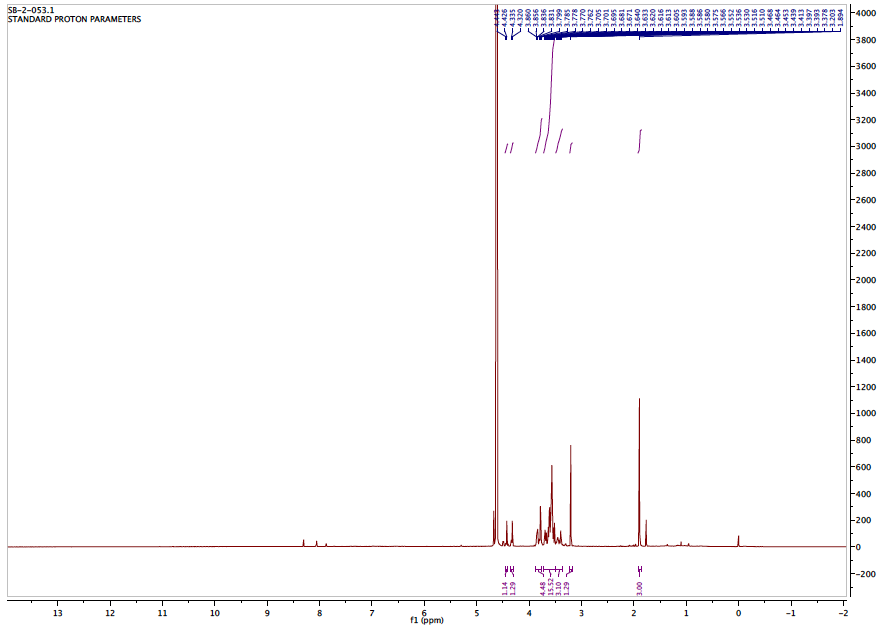


**^1^H NMR** (500 MHz) spectrum of LacNAc derivative in D_2_O

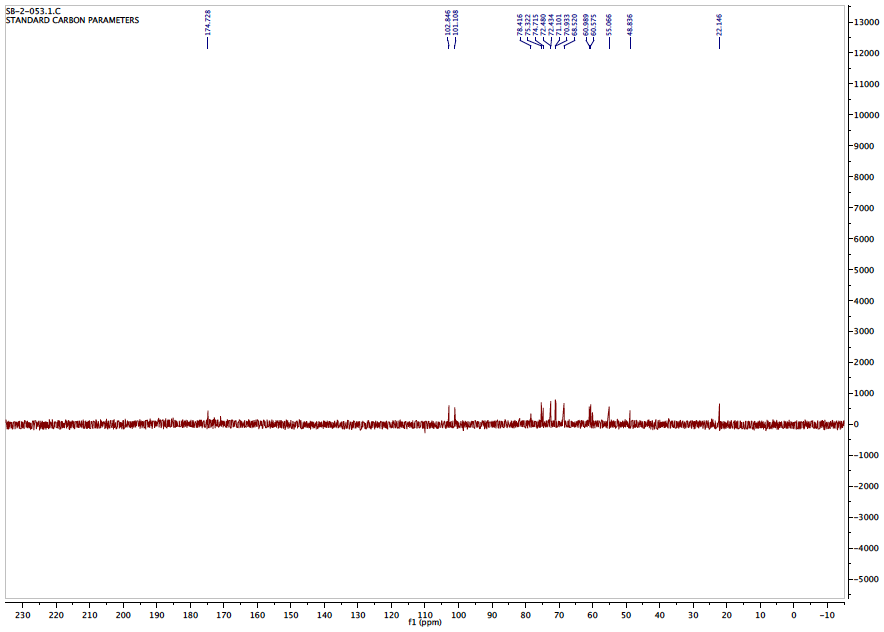


**^13^C NMR** (126 MHz) spectrum of LacNAc derivative in D_2_O
