# Supplementary material for: Structural insight into the binding of human galectins to corneal keratan sulfate, its desulfated form and related saccharides
Source: Sci Rep. 2020 Sep 24;10:15708. doi: 10.1038/s41598-020-72645-9 (PMC7515912; doi:10.1038/s41598-020-72645-9)
Supplement: Supplementary file 10 — Supplementary file10 [file 41598_2020_72645_MOESM10_ESM.docx]

**Supplemental Figure Legends**

**Supplemental Figure S1. HSQC chemical shift and broadening maps for asparagine and glutamine side chains. (A)** HSQC chemical shift map for asparagine and glutamine side chain resonances (Δδ vs. amino acid sequence) is shown for the binding of Gal-3 FL to KS at 2 μM. The horizontal black line indicates 1SD above the average value. (**B**) Resonance broadening map for asparagine and glutamine side chain resonances (ΔIntensity or ΔINT vs. amino acid sequence) is shown. A value of 1 indicates that the resonance obtained from that particular residue is no longer detectable, and a value of zero indicates no change in resonance intensity.

**Supplemental Figure S2. ^1^H NMR spectra for KS and KSDS.** Proton NMR spectra for the starting bovine KS and desulfated KS (KSDS) are shown. The clear change in chemical shifts for protons on C6 indicates successful removal of sulfate esters on the keratan polysaccharide.

**Supplemental Figure S3. PFG NMR diffusion coefficients.** Pulsed field gradient (PFG) NMR diffusion coefficients, *D*, determined using gradient-induced changes in the intensity of KS (4 μM) resonances at 3.64, 3.7 ppm are plotted as a function of the Gal-3/KS molar ratio. *D* values are shown for full-length Gal-3 (filled squares) and for the Gal-3 CRD (open circles). Solution conditions are 20 mM potassium phosphate buffer in ^2^H_2_O at pD 6.7, 30 ***◦***C.

**Supplemental Figure S4. HSQC spectra of Gal-7 bound to KS and KSDS. (A,B)** ^1^H-^15^N HSQC spectra for ^15^N-enriched Gal-7 alone (20 μM, peaks in black) and in the presence (red peaks) of keratan sulfate (KS, **A**) or desulfated KS (KSDS, **B**) at 5 μM each were overlaid. **(C,D)** ^1^H-^15^N HSQC spectra for ^15^N-enriched Gal-7 alone (20 μM, peaks in black) and in the presence (red peaks) of KS at 35 μM (**C**) or KSDS at 50 μM (**D**) were overlaid. Solution conditions are 20 mM KPhos, pH 6.9, 30 °C.

**Supplemental Figure S5. Comparisons of ligand-loaded and ligand-free Gal-3 CRD. (A)** The crystal structure of Gal-3 CRD (PDB 1A3K, colored blue) is compared to the Gal-3 CRD structure when in complex with a KS-derived tetrasaccharide following a MD simulation run and energy minimization (colored green), with some conformationally shifted residues shown in stick format as labeled. (**B**) RMSD values comparing structures shown in **A** are plotted vs. the amino acid sequence of Gal-3 CRD (residues 114 - 250). RMSD values for all atoms are shown in red, and those for backbone atoms are shown in blue. **(C)** The crystal structure of Gal-3 CRD (PDB 1A3K, colored blue) is compared to the KSDS tetrasaccharide-loaded Gal-3 CRD structure following MD simulation and energy minimization (colored green), with some conformationally shifted residues shown in stick format as labeled. (**D**) RMSD values comparing structures shown in **C** are plotted vs. the amino acid sequence of Gal-3 CRD (residues 114 - 250). RMSD values for all atoms are shown in red, and those for backbone atoms are shown in blue.

**Supplemental Figure S6. Assessment of bias from negatively charged patches on Gal-3.** (**A**) From MD simulations, the starting and final (post 50 ns) Gal-3 structures are shown for interactions with KS tetrasaccharide in orientation 1. (**B**) From MD simulations, the starting and final (post 50 ns) Gal-3 structures are shown for interactions with KS tetrasaccharide in orientation 2. For the starting structures in both orientations (**A,B**), the KS tetrasaccharide was placed near a positive patch (H223-K227, HRVKK) on the surface of Gal-3, and during these MD simulations, the KS tetrasaccharide positions do not remain stable as illustrated.

**Supplemental Figure S7. Comparisons of ligand-loaded and ligand-free Gal-9N CRD. (A)** The crystal structure of Gal-9N CRD (PDB 3WLU, colored blue) is compared to the Gal-9N CRD structure when in complex with a KS-derived tetrasaccharide following MD simulation and energy minimization (colored green), with some conformationally shifted residues shown in stick format as labeled. (**B**) RMSD values comparing structures shown in **A** are plotted vs. the amino acid sequence of Gal-9N CRD. RMSD values for all atoms are shown in red, and those for backbone atoms are shown in blue. **(C)** The crystal structure of Gal-9N CRD (PDB 3WLU, colored blue) is compared to the KSDS tetrasaccharide-loaded Gal-9N CRD structure following MD simulation and energy minimization (colored green), with some conformationally shifted residues shown in stick format as labeled. (**D**) RMSD values comparing structures shown in **C** are plotted vs. the amino acid sequence of Gal-9N CRD. RMSD values for all atoms are shown in red, and those for backbone atoms are shown in blue.

**Supplemental Material: General procedure for fluorous solid-phase extraction.** LacNAc synthesis illustrated in Scheme 1 is described in detail.
